# The transposable element environment of human genes is associated with histone and expression changes in cancer

**DOI:** 10.1186/s12864-016-2970-1

**Published:** 2016-08-09

**Authors:** Laura Grégoire, Annabelle Haudry, Emmanuelle Lerat

**Affiliations:** Université de Lyon; F-69000, France; Université Lyon 1, CNRS, UMR 5558, Laboratoire Biométrie et Biologie Evolutive, F-69622 Villeurbanne, France

**Keywords:** Transposable elements, Gene regulation, Epigenetics

## Abstract

**Background:**

Only 2 % of the human genome code for proteins. Among the remaining 98 %, transposable elements (TEs) represent millions of sequences. TEs have an impact on genome evolution by promoting mutations. Especially, TEs possess their own regulatory sequences and can alter the expression pattern of neighboring genes. Since they can potentially be harmful, TE activity is regulated by epigenetic mechanisms. These mechanisms participate in the modulation of gene expression and can be associated with some human diseases resulting from gene expression deregulation. The fact that the TE silencing can be removed in cancer could explain a part of the changes in gene expression. Indeed, epigenetic modifications associated locally with TE sequences could impact neighboring genes since these modifications can spread to adjacent sequences.

**Results:**

We compared the histone enrichment, TE neighborhood, and expression divergence of human genes between a normal and a cancer conditions. We show that the presence of TEs near genes is associated with greater changes in histone enrichment and that differentially expressed genes harbor larger histone enrichment variation related to the presence of particular TEs.

**Conclusions:**

Taken together, these results suggest that the presence of TEs near genes could favor important variation in gene expression when the cell environment is modified.

**Electronic supplementary material:**

The online version of this article (doi:10.1186/s12864-016-2970-1) contains supplementary material, which is available to authorized users.

## Background

With the advent of sequencing projects, coding genes have been revealed to correspond to a tiny fraction of eukaryotic genomes. In the human genome, the protein-coding genes represent less than 2 % of the genome, whereas repeated sequences represent more than half of it [1]. While a large fraction of the non-coding sequences was first thought to bare no function [2], it is now known to be composed of a mixture of repetitive DNA and non-functional sequences interspersed with non-coding RNA genes and regions that are crucial for transcriptional and post-transcriptional regulation [3, 4]. A large part of repeated DNA is classified as transposable elements (TEs). TEs are middle-repeated DNA sequences that have the ability to move from one position to another along chromosomes [5, 6]. These mobile elements typically encode for all the proteins necessary for their movement and possess internal regulatory regions, allowing for their independent expression. Globally, two main classes have been described according to their transposition intermediates. Retrotransposons use an RNA intermediate and form the class I, composed by the LTR-retrotransposons (endogenous retrovirus-like elements baring Long Terminal Repeat sequences on each extremity) and the non-LTR retrotransposons LINEs and SINEs (standing for Long- and Short- Interspersed Nuclear Elements respectively) that are the most frequent in the human genome [2]. Transposons use a DNA intermediate and form the class II. In the human genome, TE distribution appears to be linked to gene function. Indeed, *Alu* elements, a particular family of SINEs, were shown to be absent from the neighborhood of genes implicated in transcription and regulation [7]. Moreover, we have previously shown that TE content is associated with the function of neighboring genes: while TE-free genes are more frequently involved in development, transcription, and regulation of transcription, TE-rich genes are enriched for the functions of transport and metabolism [8].

Because of their presence in genomes, TEs have a significant impact on genome evolution by promoting various types of mutations [9, 10]. In particular, TEs possess their own regulatory sequences, and they could alter the normal expression pattern of neighboring genes while inserted in intergenic regions [11]. As an example, the MER20 element contributed to the origin of a novel gene regulatory network dedicated to pregnancy in placental mammals [12] and ERV1 elements have wired new genes into the core regulatory network of embryonic stem cells [13]. Moreover, the presence of SINEs affects the expression of neighboring genes in tumor tissue cells, with more gene deregulation associated with more SINEs in the gene vicinity [14]. In human, 0.3 % of TE insertions have been suggested for causing a disease, i.e. one insertion in every 20–100 live births [15], and approximately 96 new transposition events were directly linked to single-gene diseases [16]. Overall, the human genome harbors millions of TE insertions that could potentially affect its functioning under certain conditions. Because the effects associated with TE insertions can potentially be harmful for the host genome, TE activity needs to be regulated, a role that is partly undertaken by epigenetic mechanisms.

For the past few years, epigenetic modifications have been shown to contribute to gene expression regulation. For example, epigenetic changes can explain part of the variation in gene expression observed between tissues of a single organism [17–20], or the fate of honeybees by affecting the differentiation between the queen and the workers [21]. These examples are likely to represent only a tiny fraction of all the possible effects of epigenetic processes. Three main intertwined epigenetic mechanisms have been described so far: DNA methylation, RNA interference, and histone modifications. DNA methylation is usually occurring in the context of CpG dinucleotides in animals and is associated with transcription silencing in vertebrates [22–25]. RNA interference mechanism is characterized by the synthesis of small noncoding RNAs, which, when associated with a protein complex, can target messenger RNAs and trigger their degradation [26, 27]. Histone modifications correspond to post-translational biochemical changes occurring at particular amino acid residues of these proteins [23, 28, 29]. According to the type of histone modification, the effect can be either compacting or relaxing the chromatin structure, which have both a direct impact on gene accessibility for RNA polymerase and therefore on the gene expression [19, 30]. According to the organism, the role of each epigenetic mechanism may be more or less predominant in gene regulation. For example, DNA methylation is implicated in a large number of cellular functions in mammals and in plants, while it is almost absent from Drosophila [22, 31]. In normal condition, according to the residues and the histones, the hypermethylation of histones can be associated with methylated and repressed DNA sequences [32]. Therefore, one might expect that global alterations of histone modification patterns could disrupt gene expression. Numerous research studies have associated epigenetic changes with human diseases. For instance, cancer cells harbor global epigenetic abnormalities that could have been the initial point to tumor development [33]. For example, CpG islands, unmethylated regions overlapping the majority of human gene promoters, become hypermethylated when associated with tumor-suppressor genes, leading to their transcriptional silencing while the whole genome undergoes a global hypomethylation in cancer condition [34, 35]. Specific histone modifications, and other epigenetic processes, have been shown to specifically target TEs (for reviews, see [36, 37]). While TEs are usually methylated (and therefore silenced) in normal human cells, TE methylation is abolished in cancer cells, letting the possibility for TEs to be activated and to affect the integrity of the cell [38, 39]. For example, specific endogenous retroviruses produce viral particles in human melanoma cells [40], TE expression is enhanced in urothelial and renal carcinoma cells [41], in some carcinomas [42], in human leukemia [43, 44], and in human colorectal, ovarian and breast cancers [45–48]. These activations are potentially resulting from different epigenetic modifications occurring in a cancer cell. The majority of the studies concerning the epigenetic alterations occurring on TEs in a cancer environment have mainly focused on DNA methylation (for a review see [49]). While only a few studies investigated TE histone modifications, a global loss of monoacetylation of lysine 16 and of trimethylation of lysine 20 on histone 4 has been found associated to repetitive elements [50]. Moreover, the spread of TE histone modifications to adjacent regions has been observed in plants, fungi, and mouse [51–54] suggesting that the presence of TEs may influence the epigenetic state of neighboring genes. Among the different mechanisms that could explain the effects of epigenetic changes in a cancer cell, the implication of TE insertions, harmless in normal conditions but for which epigenetic changes could lead to a cascade of deregulation either causing or reinforcing the tumor status of a cell, still needs to be investigated.

Here, we first observed the variation of ten histone modifications and TE content of genes according to their genomic position in normal condition. We observed that genes are generally more enriched in activating modifications at all chromosome locations compared to repressive modifications. We then compared the histone modification landscapes of genes in normal and cancer blood cell lines, according to their TE neighborhood. Our results showed that the presence of TEs near human genes is associated with greater changes in histone enrichment. Finally, we could highlight that differentially expressed human genes harbored larger histone enrichment variation related to the presence of TEs. Taken together, these results suggest that the presence of TEs near genes could favor important variation in gene expression when the cell environment is modified in human.

## Methods

### Data acquisition

Gene locations were downloaded from the Biomart server using the Martview tool [55] (www.ensembl.org/biomart/martview/) on the last version of the human genome (GRCh37.p10 = hg19). Over a total of 62,380 genes in the human genome, we filtered for protein coding genes located on the 22 autosomal and the two sexual chromosomes, removing those located on the mitochondrial genome and unidentified chromosomes, and retrieved 19,071 genes. For each gene, Ensembl identification number, strand orientation, and localization (start and end positions on the chromosome) were collected.

TE insertions in human genome were previously identified using RepeatMasker [56], a program that determines the occurrences of sequences with homology to consensus TE sequences present in the Repbase database [57] and were retrieved from the website of the University of California, Santa Cruz (ftp://hgdownload.cse.ucsc.edu/goldenPath/hg19/chromosomes/). The RepeatMasker output files were parsed using the program “One code to find them all” [58] (with the --*strict* option) to assemble each TE copy and determine their localization.

Locations of histone modifications produced by ChIP-seq experiments were downloaded for the last version of the human genome on the ENCODE Genome Browser (http://hgdownload.cse.ucsc.edu/goldenPath/hg19/encodeDCC/wgEncodeBroadHistone/). They correspond to broader regions of enrichment (broadPeaks) [59]. These regions were retrieved for 10 histone modifications (H3K4me1, H3K4me2, H3K4me3, H3K9ac, H3K9me3, H3K27ac, H3K27me3, H3K36me3, H3K79me2, and H4K20me1) and for two different conditions: a lymphoblastoid cell line originated from normal peripheral blood lymphocyte of a female donor (GM12878 named “normal condition”) and a leukemic cell line originated from derived from a female patient with chronic myeloid leukemia (K562 named “cancer condition”). The two replicates of expression data obtained by RNA-seq experiments were retrieved for the two different conditions (GM12878 and K562) on the ENCODE Genome Browser (http://hgdownload.cse.ucsc.edu/goldenPath/hg19/encodeDCC/wgEncodeCaltechRnaSeq/).

### Mean histone enrichment for each gene

To determine the mean histone enrichment of each gene for a given histone modification, we computed the average fold enrichment *ε* of the histone modifications for the positions covered by an entire gene, normalized by the gene size (E1). We chose not to focus only on the promoter region since it has been shown that some of the modifications can be enriched also along the transcribed region of a gene with very different levels of enrichment between active and inactive genes [60, 61].

1 $$ \varepsilon (h)=\frac{\sum {e}_i}{n\ast l} $$

with *h* the histone modification, *n* the number of values of fold enrichment of the histone modification *h* mapped within the gene, *e*_*i*_ the value of enrichment of the histone modification *h* at position *i* mapped within the gene, and *l* the length of the gene.

### Computation of the density and coverage of TEs in the vicinity of genes

To estimate the amount of TEs within and around genes, we first used each TE position to allocate it to a gene vicinity, using a 2 kb-flanking region upstream, to include gene promoters, and downstream the gene [8]. Then, for each gene, the density in TEs reported as the number of insertions per base pair (E2) and the coverage in TEs, in percentage of the gene (E3), were computed in general for all TEs and for each TE type (DNA transposons, LTR-retrotransposons, LINEs, and SINEs).

2 $$ {D}_g=\frac{N}{L_g-{L}_{\mathrm{TE}}} $$

3 $$ {C}_g=\frac{L_{\mathrm{TE}}}{L_g} $$

with *g* the gene, N the number of TEs, *L*_*g*_ the length of the gene plus its 2 kb-flanking region, and *L*_*TE*_ the number of nucleotides annotated as TEs in the region encompassing the given gene.

These two different metrics were used because the number of TEs associated with a gene is affected by the size of the gene and its flanking region, and by its own size. Whereas the density rather estimates the number of insertions, the coverage measures the proportion of nucleotides belonging to an element in the sampled sequence. The relationship between these two statistics was tested by a Spearman correlation test.

Genes were clustered according to their level of density and coverage of TEs using the *pam()* function of the R package [62]. This algorithm, called “Partitioning Around Medoids”, provides a robust clustering method because outliers have a less important impact than in the *k-means* method often used for clustering [63]. The main difference between the two methods is that *pam()* uses a minimization of dissimilarities instead of a sum of Euclidean distances, and that the medoids (center of a cluster) is an actual point within the dataset. The genes with density and coverage equal to 0 were defined as TE-free genes (4,300 genes). The remaining 14,771 genes were clustered with the *pam()* function to discriminate between the TE-intermediate (9,132 genes) and the TE-rich genes (5,639 genes). To have more precise information concerning the influence of particular TE types, we also classified the 14,771 genes according to the density and coverage for each particular TE type using the *pam()* function. We thus determined 11 different categories: the all-TE-intermediate and all-TE-rich categories that correspond to genes with respectively intermediate and rich levels for every TE types, and the SINE-rich, LINE-rich, DNA-rich, LTR-rich, SINE-intermediate, LINE-intermediate, DNA-intermediate, LTR-intermediate, plus a “mix” category, which contains genes with a combination of TE types. To avoid any confounding factors due to the simultaneous presence of different types of TE near genes, we applied a strict rule to determine the category. For example LINE-intermediate genes are free from other TE types.

### Differential gene expression and functional analyses by GO term enrichments

RNAseq reads from both samples GM12878 and K562 were trimmed to ensure sequencing quality using the unsupervised approach of the program *UrQt* [64] and aligned against human genes using *Tophat2* [65]. Alignment counts were obtained on sorted bam files using *htseq-count* [66], and differential gene expression was assessed using *DESeq2* [67]. We used an adjusted p-value threshold <0.1 for significance, which allowed us to identify 7,724 genes differentially expressed over the 19,071 total protein coding genes. We determined the enrichment in particular GO terms in a list of target genes (for example down-regulated genes in cancer condition) by comparing it with the list of all the genes in the genome using GOrilla [68] and REVIGO [69].

### Statistical analyses

All statistical analyses were performed using the R software [62]. To account for multiple testing and to be conservative, we used the Bonferroni correction and considered significant the results with *p* values < 0.05/n, n being the number of tests realized.

## Results

### Histone modifications and TE enrichment of genes vary according to the gene position on chromosome

**Fig. 1:**
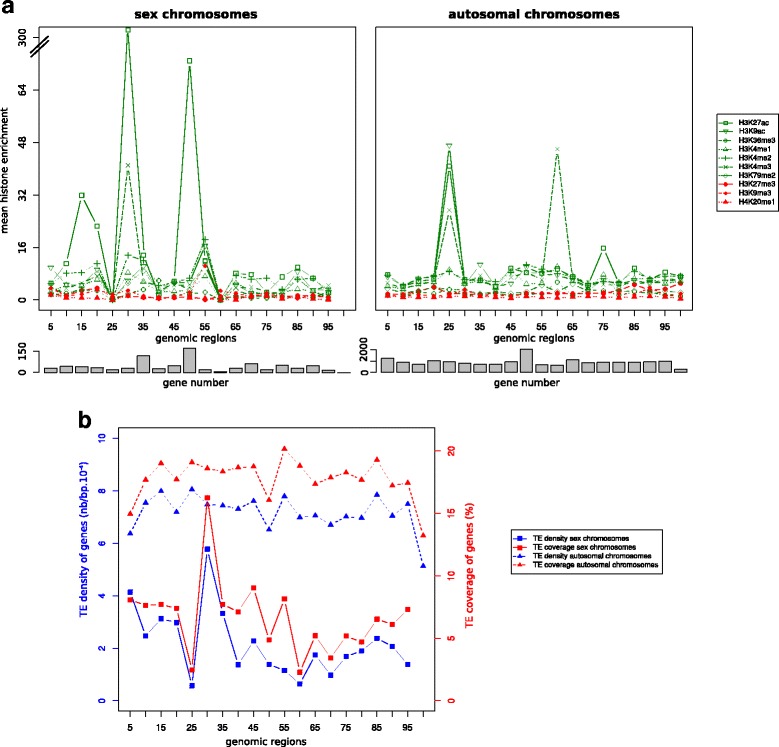
**a**. Distribution of the mean histone enrichment along sex and autosomal chromosomes for the 10 histone modifications in the normal condition (GM12878). **b** Distribution of the TE density and TE coverage of genes along sex and autosomal chromosomes

We observed the mean histone enrichment of genes according to gene position on chromosomes in normal condition (Fig. 1a, Additional file 1 for each chromosome). We split each chromosome in bins representing 5 % of the total chromosome length, i.e.*,* genes located in terminal regions of the chromosomes are located in bins 5 % and 100 %. Independently of the chromosome location and for both sex and autosomal chromosomes, genes are on average less enriched for repressive histone modifications than for activating histone modifications. However, there are some local variations according to the histone modification. On sex chromosomes, H3K27ac is particularly enriched at four locations. In each case, this is due to a small subset of genes that display particularly high enrichment for this modification (Additional file 2). Some of these genes are also responsible for the peak corresponding to a high level of enrichment for H3K4me3. Less important peaks of mean enrichment are also observed on autosomal chromosomes for three locations, which concern the same histone modifications in addition to H3K9ac (Additional file 3).

We also observed the variation in TE density and TE coverage of genes according to their location on chromosomes. As both metrics are highly correlated (*r* = 0.95, *p* < 2.2e-16), either of them can be used to determine the TE richness of each gene vicinity. Globally, TE density and TE coverage values tend to be lower for genes located on sex chromosomes than for autosomal genes (Fig. 1b, Additional file 4 for each chromosome). Moreover, the level of variation in TE density and TE coverage of genes is more important for genes located on sex chromosomes than for autosomal. Especially, genes located on the bin 30 % of the sex chromosomes display a higher TE density and coverage than the genes from the other part of these chromosomes.

### The presence of TEs is locally associated with greater changes in the chromatin environment of genes between normal and cancer conditions

**Fig. 2:**
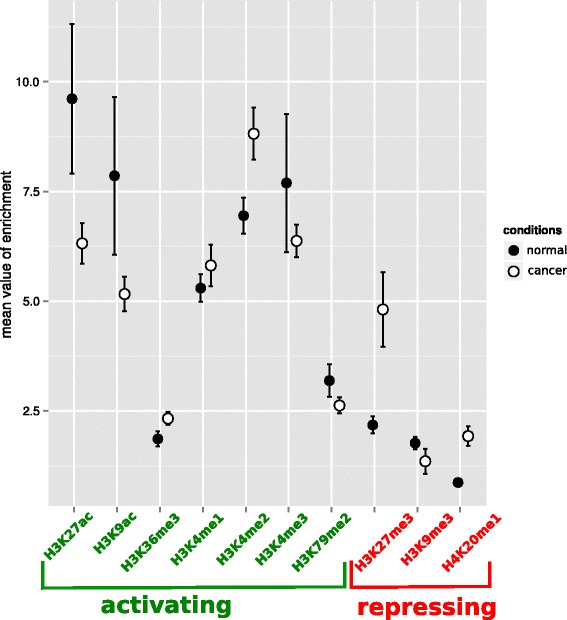
Mean histone enrichment of genes for the 10 histone modifications in the two conditions: normal (GM12878) and cancer (K562). The modifications known to participate in the expression of genes or to be associated with open chromatin are represented in green. Those known to induce gene repression or to be associated with closed chromatin are represented in red. Vertical bars indicate the mean +/− standard errors

We determined how the histone enrichment of genes varies between the two conditions, normal and cancer. There is no clear general pattern of enrichment or depletion in activating modifications associated with cancer (Fig. 2). However, except the activating modification H3K79me2, all modifications display different profiles of enrichment between the two conditions (Wilcoxon paired tests, *p* < 0.005). For example, genes are on average more enriched in H3K27ac in normal condition compared to the cancer condition, when it is the reverse for the H3K27me3 modification.

**Fig. 3:**
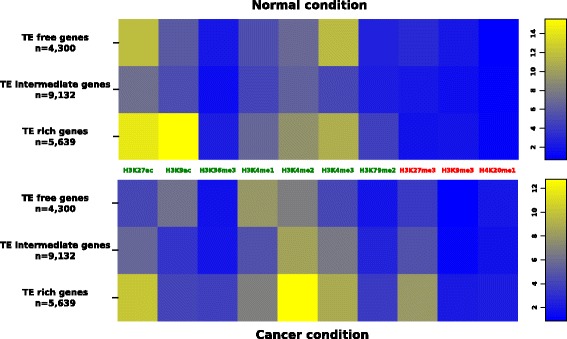
Heatmap of the mean enrichment for the 10 histone modifications of genes according to the TE category of their neighborhood in the two conditions: normal (GM12878) and cancer (K562). The number of genes of each category is given (n). High enrichments are toward yellow color whereas low enrichments are toward dark blue color

To determine if the presence of TEs near genes may be associated with greater changes in histone modifications of genes between the two conditions, we computed the mean histone enrichment for the genes according to their TE category: TE-free, TE-intermediate or TE-rich (Fig. 3). For each condition, we found that some histone modification enrichments vary when comparing TE-rich and TE-free genes (Additional file 5; Wilcoxon tests, *p* < 1.67e-3). For example, in normal condition, TE-rich genes are more than twice enriched for H3K9ac than TE-free genes (ε_H3K9ac_ = 15.49 and 6.01 respectively, *p* < 2.2e-16). We then compared the histone enrichment for each gene between the two conditions and we observed that excepted for H3K79me2 in all gene categories and for H3K27ac in TE-free and TE-rich genes, the histone enrichment is different between the two conditions inside each gene category (Wilcoxon paired tests, *p* < 8.3e-4). TE-rich genes are more enriched in H3K9ac in normal condition than in cancer condition (ε_H3K9ac_ = 15.49 and 7.98 respectively, *p* < 2.2e-16). However, TE-rich genes are more enriched in H3K4me2 and H3K27me3 in cancer condition (ε_H3K4me2_ = 12.72 and ε_H3K27me3_ = 4.13) compared to the normal condition (ε_H3K4me2_ = 9.15 and ε_H3K27me3_ = 1.87, *p* < 2.2e-16 and *p* < 2.2e-16 respectively).

**Fig. 4:**
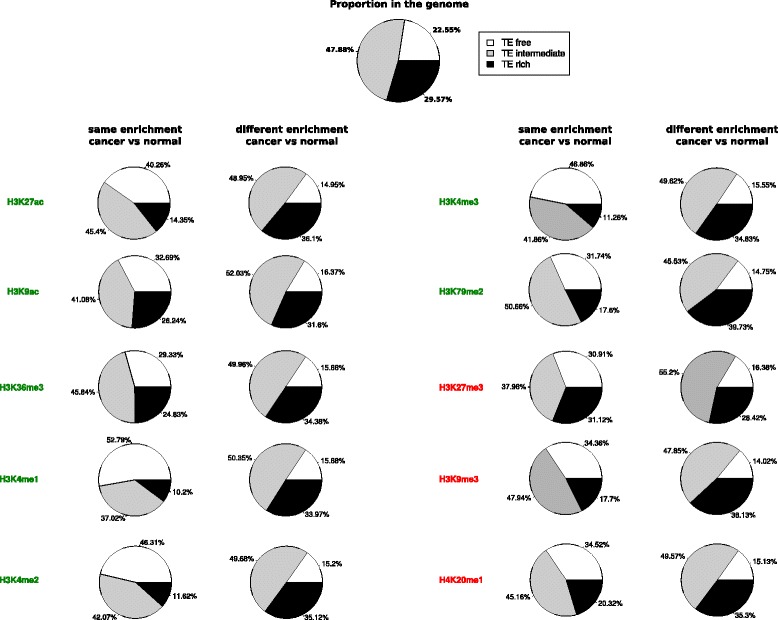
Gene proportion according to the TE category of their neighborhood. The gene proportion is shown for the global genome and between the two conditions (normal (GM12878) and cancer (K562)) for genes displaying the same histone enrichment and for genes displaying different histone enrichment for the 10 histone modifications

The previous analyses showed that histone enrichment does vary according to the TE content in the neighborhood of genes. However, it is not expected that particular levels of enrichment could be systematically associated to the presence or absence of TEs. We tested whether the presence of TEs is associated with a greater variation in histone enrichment between the two conditions, whatever the level of enrichment. To determine any over or under-representation of each gene category according to their proportion in the genome, we compared their number to (*i*) the number of genes displaying similar enrichment in normal and cancer conditions, and (*ii*) the number of genes displaying significantly different enrichment between the two conditions. The results are presented on Fig. 4. Chi2 homogeneity tests showed that distribution of the number of genes from each TE-content category is significantly different when considering variation in histone enrichment compared to their distribution in the whole-genome (*p* < 0.0025). Globally, the TE-free genes are more frequently showing similar histone modification enrichment in the two conditions, while TE-rich genes tend to exhibit differences. For example, the genes without variation in histone enrichment between normal and cancer conditions for H3K4me1 and H4K20me1 are more represented by TE-free genes compared to their proportion in the genome (respectively 52.79 and 34.52 %, instead of 22.55 %). For the same histone modifications, in the genes that exhibit different histone enrichment between normal and cancer conditions, the proportion of TE-free genes decreases (15.68 % or H3K4me1, and 15.13 % for H4K20me1) whereas the proportion increases for the TE-intermediate (50.35 % for H3K4me1 and 49.57 % for H4K20me1) and TE-rich genes (33.97 % for H3K4me1, and 35.30 % for H4K20me1). Taken together, these results indicate that a gene with TEs in its vicinity is more likely to have a change in histone enrichment between the two conditions compared to a TE-free gene.

**Fig. 5:**
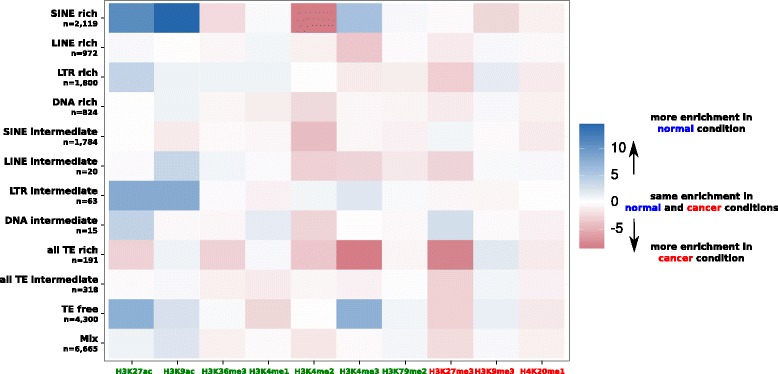
Differential histone enrichment between normal (GM12878) and cancer (K562) condition for the 10 histone modifications of genes according to the TE category of their neighborhood. The number of genes of each category is given (n). More enrichments in normal condition are toward blue color whereas more enrichments in cancer condition are toward read color. White color corresponds to an absence of differential enrichment between the two conditions

In some particular cases, TEs can be associated with various histone modifications according to their classes [70, 71]. To determine if similar patterns were found when considering TE types individually, we computed the mean differential enrichment of genes between normal and cancer conditions according to the TE type in the gene neighborhood for each histone modification (Fig. 5 and Additional file 6). The presence of different types of TEs near genes is associated with different effects (Kruskal Wallis, tests *p* < 0.005). In particular, SINE-rich, LTR-intermediate, and TE-free genes are more enriched for H3K4me3 in normal condition, whereas LINE-rich, LINE-intermediate, and all-TE-rich genes are more enriched for this modification in cancer condition.

### Differentially expressed genes between normal and cancer conditions have particular histone enrichment variations and TE environment

To test a possible association between the presence of TEs, particular histone enrichment, and gene expression, we analyzed in more detail the 7,699 genes differentially expressed between the two conditions for which histone modifications were associated, the 25 missing genes being located on unidentified chromosomes. Down-regulated genes in the cancer condition compared to normal one are enriched for functions in the regulation of lymphocyte activation, the defense response, and the immune system process. Up-regulated genes are enriched for functions in cytoskeleton organization, cell cycle process, sulfur compound biosynthesis, regulation of vesicle mediated transport, single organism cell process, and post-translational protein folding (Additional file 7). We have also compared our datasets of down- and up-regulated genes to the set of census cancer genes identified in the COSMIC database (http://cancer.sanger.ac.uk/cosmic; [72]). The results show that among the 596 census genes that have been identified as “cancer genes”, meaning genes for which mutations have been causally implicated in cancer, 156 and 120 correspond to genes from our sets of down- and up-regulated genes respectively.

The mean histone enrichment of up- and down-regulated genes in cancer condition in comparison to the normal one is reported in Table 1, for both conditions. The histone enrichment is significantly different between the two conditions for all modifications, and for up- and down-regulated genes (Wilcoxon paired tests, *p* < 0.0025) with the only exceptions of H3K4me2 for down-regulated genes and H3K27me3 for up-regulated genes. Both up- and down-regulated genes display the same pattern with more enrichment in normal condition for H3K27ac, H3K36me3, H3K9me3, H3K9ac, and more enrichment in cancer condition for H4K20me1. It is therefore unlikely that the divergence of expression in response of the cancer is due to these modifications. However, up-regulated genes are more enriched for H3K4me1, H3K4me2, H3K4me3, and H3K79me2 in cancer condition whereas the down-regulated genes are depleted for these activating modifications in the same condition (except for H3K4me2, which displays no difference between the normal and cancer conditions). Symmetrically, down-regulated genes are more enriched in cancer condition for the repressive histone modification H3K27me3 whereas up-regulated genes do not show variation between the two conditions. These differences could potentially explain the divergence of expression of these genes between the two tested conditions. In order to determine if some particular functions could be more represented among these genes, we looked at the Gene Ontology terms of the most highly down-regulated genes that are TE-rich and enriched in H3K27me3 in cancer condition (Additional file 8). Interestingly, seven out of the 15 genes are implicated in immune system process and response to stress, among which one gene, LCK, is identified as a “cancer gene” in the COSMIC database. Similarly, we looked at the most highly up-regulated genes that are either TE-intermediate or TE-rich, and enriched in H3K79me2 (Additional file 9). In that case, there is less common GO terms but we can notice that among the 43 genes, six are involved in immune system process and response to stress, and four are involved in transcription from RNA polymerase II. Among the genes from this last category, two have been identified as “cancer genes” in the COSMIC database (GATA1 and GATA2).

**Table 1:** Mean histone enrichment for the 10 histone modifications of genes according to their expression divergence between normal and cancer condition

		Down-regulated genes			Up-regulated genes		
	Histone modification	Normal	Cancer	Wilcoxon paired tests *p*-values	Normal	Cancer	Wilcoxon paired tests *p*-values
activating	H3K27ac	8.05^*^	7.35	<2.2e-16	13.46^*^	9.11	<2.2e-16
	H3K9ac	8.63^*^	5.92	<2.2e-16	7.66^*^	7.39	2.78e-13
	H3K36me3	2.91^*^	2.49	<2.2e-16	2.49^*^	1.65	<2.2e-16
	H3K4me1	5.58^*^	5.28	8.99e-5	6.09	6.28^*^	<2.2e-16
	H3K4me2	9.74	9.51	0.1682	8.06	11.10^*^	<2.2e-16
	H3K4me3	6.83^*^	5.73	0.0002763	6.65	9.42^*^	<2.2e-16
	H3K79me2	4.03^*^	1.92	<2.2e-16	2.97	4.46^*^	<2.2e-16
repressing	H3K27me3	1.76	5.86^*^	<2.2e-16	3.54	3.35	0.009098
	H3K9me3	2.16^*^	0.96	<2.2e-16	1.69^*^	1.40	3.46e-7
	H4K20me1	1.27	1.52^*^	5.56e-9	0.92	2.97^*^	<2.2e-16

^*^Significantly more enrichment (*p*-value < 0.0025)

The TE environment appears to be associated with the variation in histone modifications observed between the up- and down-regulated genes (Table 2). Among the differentially expressed genes displaying enrichment or depletion in particular histones, we tested whether the number of genes regarding their local TE landscape is different from that observed in the total genome. We first considered the down-regulated genes with more enrichment in H3K27me3 in cancer condition (1,514 genes) and depleted in H3K4me1 (1,649 genes), H3K4me3 (1,420 genes), and/or H3K79me2 (1,766 genes). Globally, the proportions are different for all comparisons (Chi2 homogeneity tests, *p* < 0.0055). More specifically, there is an increase of LTR-rich genes inside each group of genes (9.44 % (total genome) versus 17.97 % (H3K27me3), 15.46 % (H3K4me1), 14.37 % (H3K4me3), and 16.08 % (H3K79me2)) whereas the proportion of TE-free genes greatly decreases (22.55 % (total genome) versus 11.23 % (H3K27me3), 14.55 % (H3K27me3), 15.56 % (H3K4me3), and 12.85 % (H3K79me2)). We also observe an increase in the proportion of DNA-intermediate genes (0.08 % (total genome) versus 0.13 % (H3K27me3) and 0.14 % (H3K4me3)), all-TE-intermediate genes (1.67 % (total genome) versus 2.77 % (H3K27me3)), and all-TE-rich genes (1.00 % (total genome) versus 2.46 % (H3K4me3) and 2.38 % (H3K79me2)), but also a decrease in the proportions of SINE-rich, SINE-intermediate, and LTR-intermediate genes. Among the up-regulated genes that display enrichment in H3K4me1 (2,334 genes), H3K4me2 (2,345 genes), H3K4me3 (2,583 genes), and/or H3K79me2 (1,819 genes), the proportions of SINE-rich, DNA-intermediate, and LTR-rich genes increase whereas the proportions of LINE-intermediate, LTR-intermediate, and TE-free genes decrease.

**Table 2:** Gene number (proportion) among differentially expressed genes according to the TE-content category and their enrichment in histone modifications in cancer condition

TE category	Total genome	Down-regulated genes				Up-regulated genes			
		depleted in H3K4me1	depleted in H3K4me3	depleted in H3K79me2	enriched in H3K27me3	enriched in H3K4me1	enriched in H3K4me2	enriched in H3K4me3	enriched in H3K79me2
SINE-rich	2119 (11.11 %)	156 (9.46 %)	146 (10.28 %)	161 (9.12 %)	114 (7.53 %)	298 (12.77 %)	292 (12.45 %)	310 (12.00 %)	227 (12.48 %)
LINE-rich	972 (5.10 %)	88 (5.34 %)	82 (5.77 %)	94 (5.32 %)	80 (5.28 %)	96 (4.11 %)	107 (4.56 %)	111 (4.30 %)	78 (4.29 %)
DNA-rich	824 (4.32 %)	91 (5.52 %)	74 (5.21 %)	87 (4.93 %)	73 (4.82 %)	96 (4.11 %)	101 (4.31 %)	97 (3.76 %)	77 (4.23 %)
LTR-rich	1800 (9.44 %)	255 (15.46 %)	204 (14.37 %)	284 (16.08 %)	272 (17.97 %)	247 (11.58 %)	238 (10.15 %)	267 (10.34 %)	209 (11.49 %)
SINE-intermediate	1784 (9.35 %)	118 (7.16 %)	111 (7.82 %)	98 (5.55 %)	73 (4.82 %)	229 (9.81 %)	236 (10.06 %)	250 (9.68 %)	177 (9.73 %)
LINE-intermediate	20 (0.10 %)	0 (0.00 %)	1 (0.07 %)	1 (0.06 %)	2 (0.13 %)	1 (0.04 %)	1 (0.04 %)	0 (0.00 %)	1 (0.05 %)
DNA-intermediate	15 (0.08 %)	1 (0.06 %)	2 (0.14 %)	1 (0.06 %)	2 (0.13 %)	3 (0.13 %)	4 (0.17 %)	2 (0.08 %)	3 (0.16 %)
LTR-intermediate	63 (0.33 %)	7 (0.42 %)	5 (0.35 %)	6 (0.34 %)	1 (0.07 %)	4 (0.17 %)	5 (0.21 %)	3 (0.12 %)	3 (0.16 %)
TE-free	4300 (22.55 %)	240 (14.55 %)	221 (15.56 %)	227 (12.85 %)	170 (11.23 %)	404 (17.31 %)	407 (17.36 %)	454 (17.58 %)	314 (17.26 %)
All-TE-intermediate	191 (1.67 %)	14 (0.85 %)	6 (0.42 %)	20 (1.13 %)	16 (2.77 %)	41 (1.76 %)	40 (1.71 %)	44 (1.70 %)	37 (2.03 %)
All-TE-rich	318 (1.00 %)	27 (1.64 %)	35 (2.46 %)	42 (2.38 %)	42 (1.06 %)	25 (1.07 %)	30 (1.28 %)	32 (1.24 %)	18 (0.99 %)
Mix	6665 (34.95 %)	652 (39.54 %)	533 (37.53 %)	745 (42.19 %)	669 (44.19 %)	890 (38.13 %)	884 (37.70 %)	1013 (39.22 %)	675 (37.11 %)
P values Chi2 homogeneity tests		<2.2e-16	6.863e-14	<2.2e-16	<2.2e-16	<2.2e-16	2.208e-06	3.434e-09	5.405e-06
Total gene number	19071	1649	1420	1766	1514	2334	2345	2583	1819

Chi2 homogeneity tests to compare the gene number for each modification to the gene number in total genome *p* < 6.25e-3

## Discussion

In this work, we showed that genes are generally more enriched for activating histone modifications than for repressive ones when considering all positions on chromosomes, in both autosomal and sex chromosomes. This may reflect the fact that genes are usually enriched in regions associated to an open chromatin state [73, 74]. We did not detect any significant effect of the local gene density on a chromosome on the histone modification enrichment pattern (Spearman correlation tests, data not shown). However, at a more fine scale, we know that variations among genes exist according to their function in the tissue considered. We observed regions with high level of enrichment for activating histone modifications, which are due to especially high values associated to a small number of genes. This could point to genes particularly active in the analyzed cell line since it has been shown that histone modification levels are good predictors of the gene expression level [75]. When we analyzed the TE content near genes, we observed that genes are on average more enriched in TEs when located on autosomal chromosomes when compared to genes present on sex chromosomes. This is in general agreement with previous analyses made on the TE distribution in the human genome, where the density of some retroelements is higher on autosomal chromosomes than on the X chromosome [76], which could be associated with variation in the recombination rate on these chromosomes.

We did not observe any general pattern of increase or decrease of histone modifications according to their effect on gene expression in association with cancer compared to the normal state, but the two conditions showed significantly different landscapes for enrichment. Variances of enrichment for some histone modifications appear to be larger for genes in normal condition. This points out the need to better understand how epigenetic modifications are labile to quantify how much they vary among normal conditions, across time, or even among individuals, a whole body of research that is just starting [77]. For the purpose of the study, we made the hypothesis that the “within condition” variation can be estimated using the large number of genes corresponding to the whole-genome.

Our results showed that there is more variation in the histone enrichment of genes between normal and cancer condition, when the genes are enriched in TEs. This could be linked to the fact that TEs can be associated to particular epigenetic modifications. In human and mouse, TEs are associated with H3K9me3 and H4K20me3 [78, 79]. In mouse, an association of the modification H3K27me3 to SINEs and gene rich regions has been shown [80]. Histone modifications play a major role in the global silencing of TEs in the mammal genomes, even if some variability exists regarding the TE family [78, 79, 81, 82]. Interestingly, some of the histone modifications are likely to be cell-type specific and could indicate that some of them targeting TEs may regulate the expression of “host” genes, especially if they provide the host with a function [82]. Particular histone modifications of TEs have also been shown to spread to the neighboring regions of the TE insertion. For example, Intracisternal *A-particle* (IAP) elements, which are moderately repeated TEs in mouse (~1000 copies) induce H3K9me3 and H4K20me3 targeting on flanking regions of their insertion [54]. A similar observation has been made in plants, in which the insertions of TEs in euchromatic regions induce the local formation of heterochromatin [53, 81, 83]. Hence, the presence of particular histone modifications associated with TEs could influence the epigenetic profile of neighboring genes, due to the synergetic or antagonist actions of different histone modifications [84]. In cancer condition, the global modifications occurring on TEs may also spread to neighboring genes inducing changes in their expression, which in turn would perturb various genetic networks. Indeed, in cancer cells, silencing of tumor-suppressor genes by hypermethylation of CpG island promoters is associated with deacetylation of histones H3 and H4, loss of H3K4me3, and gain of H3K9me and H3K27me3 [35, 85]. However, unmethylated tumor-suppressor genes are silenced when hypoacetylation and hypermethylation of histones H3 and H4 are present, indicating that only changes of histone modifications can be sufficient to repress a gene [34]. A global reduction of monoacetylated H4K16 has been observed in cancer cells, along with a loss of the active modification H3K4me3 and of the repressive modification H4K20me3, and a gain of the repressive modification H3K27me3 [50, 85, 86]. Interestingly, we did not observe an association with more repressive histone modifications for TE-rich genes compared to TE-free genes in normal condition, as could be expected if all TE insertions are indeed only targeted by silencing modifications. Some of the TE insertions might have been selected for their adaptive role in the gene regulation, and therefore not silenced by the host-genome. A theory concerning an “exaptation hypothesis” has been suggested [87]. The authors proposed that the role of TE epigenetic modifications could be adaptive, with TEs having been recruited to participate in the regulation of host genes, although some evidences remained in support to the alternative hypothesis of “genome defense”, in which epigenetic regulatory system evolved to silence TEs and prevent their deleterious activities. In any case, this implies that among all TE insertions in a genome, not all of them will have the same impact on gene expression, according to their impact on natural selection.

Among the differentially expressed genes between the two conditions and presenting variation in histone enrichments, genes with particular TEs in their vicinity are over-represented while TE-free genes are under-represented. This was especially clear for down-regulated genes. This result supports a causal link between the presence of TEs, the histone modifications and the changes in gene expression. In cancer condition, epigenetic remodeling of large genomic region is observed, as well as a loss of control of various epigenetic mechanisms [88, 89]. The presence of TEs in these regions could thus trigger particular changes in epigenetic modifications when compared to regions devoid of TEs. Interestingly, the effect seems to change according to the type of TEs present near genes. We showed here that the proportion of LTR-rich genes increases among down-regulated genes with a depletion in several activating histone modifications and an enrichment in the repressive modification H3K27me3 in cancer condition. Similarly, an effect on gene expression has been observed for L1 elements when inserted into genes, associated with DNA hypomethylation in cancer condition [90]. In addition, we observed that LINE-intermediate and LINE-rich genes are less represented among up-regulated genes in cancer condition, which could be linked to the same effect.

In this study, we have made the hypothesis that all TE insertions currently present in the human genome are fixed. Although it is true for the large majority of the millions of insertions of this genome, a small number of TE families corresponding to non-LTR retrotransposons are known to be still active and potentially able to produce new insertions, which corresponds to a few thousand active copies [16, 91]. Since in cancer conditions more transcriptional activity of TEs has been observed, new insertions could be generated for the families still active. Some studies have indeed identified several hundred of somatic transposition events in various cancer tissues that were mainly found inside known cancer genes, indicating a direct link between the new insertions and the cancer development [92–95]. Novel insertions may provide particular changes in the epigenetic profiles of genes inside or near which they insert that we would not be able to detect here. However, it would not completely change the global pattern we observed since these new insertions cannot change completely the TE category of genes, except for some of the TE-free genes. Moreover, since we focused on genes having one category of TE in their neighborhood to avoid confounding factors of various TE families, it is unlikely that new insertions would be inserted in the genes we considered. Although new cancer insertions may not blur the observations we made, the use of polymorphic insertions would be especially interesting to directly measure the influence on gene expression and epigenetic modifications according to the differential presence / absence of active TEs near particular genes. For example, the study of paralogous regions in the human genome has shown that the presence of *Alu* elements is associated with DNA methylation divergence, with a hypermethylated region being closer to *Alu*s than to their corresponding hypomethylated copy [96]. Then the differential presence of some TE insertions could in some cases be associated with variation in the epigenetic landscape of genes, which may be associated to certain susceptibility to cancer development. These polymorphic insertions have been shown to be more numerous than somatic cancer insertions since they can represent a few thousand sequences [16, 92, 97]. However, these insertions are usually not found near genes, as a consequence of the direct action of natural selection, which eliminates deleterious mutations. Then, it can be expected that not having considered these insertions would not modify our current results.

## Conclusions

Our analyses have shown that the genomic environment of genes is important to understand changes in gene expression when the cell undergoes changes of condition. The presence of TEs around genes may have crucial impact on their epigenetic landscape.

## Abbreviations

GO, Gene Ontology; LINEs, Long Interspersed Elements; LTR, Long Terminal Repeat; SINEs, Short Interspersed Nuclear Elements; TEs, transposable elements

## Additional files

Additional file 1: Figure S2. Distribution of the TE density and TE coverage of genes along all chromosomes. (PDF 311 kb) Additional file 2: Table S1. Mean enrichment of genes responsible for the high mean enrichment value for H3K27ac and/or H3K4me3 at particular positions of sex chromosomes. (PDF 207 kb) Additional file 3: Table S2. Mean enrichment of genes responsible for the high mean enrichment value for H3K27ac H3K4me3 and/or H3K9ac at particular positions of autosomal chromosomes. (PDF 260 kb) Additional file 4: Figure S1. Distribution of the mean histone enrichment along all chromosomes for the 10 histone modifications in the normal condition (GM12878). The chromosome Y is not represented since all of its 45 genes did not present any histone enrichment values. (PDF 252 kb) Additional file 5: Table S3. Mean values of histone enrichment for each histone modification and for each gene category. (PDF 231 kb) Additional file 6: Table S4. Differential mean values of enrichment for each histone modification and for each gene category according to the TE type. (PDF 31 kb) Additional file 7: Table S5. GO function of down- and up-regulated genes between normal and cancer condition. (XLSX 870 kb) Additional file 8: Table S6. Most highly down-regulated TE-rich genes that are enriched for H3K27me3 in cancer condition (PDF 206 kb) Additional file 9: Table S7. Most up-regulated TE-rich and TE-intermediate genes that are enriched for H3K79me2 in cancer condition (PDF 213 kb)

## References

[1] International Human Genome Sequencing Consortium (2004). Finishing the euchromatic sequence of the human genome. Nature.

[2] Lander ES, Linton LM, Birren B, Nusbaum C, Zody MC, Baldwin J, Devon K, Dewar K, Doyle M, FitzHugh W (2001). Initial sequencing and analysis of the human genome. Nature.

[3] Cordaux R, Batzer MA (2009). The impact of retrotransposons on human genome evolution. Nat Rev Genet.

[4] Ludwig M (2002). Functional evolution of noncoding DNA. Curr Opin Genet Dev.

[5] Wicker T, Sabot F, Hua-Van A, Bennetzen JL, Capy P, Chalhoub B, Flavell A, Leroy P, Morgante M, Panaud O (2007). A unified classification system for eukaryotic transposable elements. Nat Rev Genet.

[6] Kapitonov VV, Jurka J (2008). A universal classification of eukaryotic transposable elements implemented in Repbase. Nat Rev Genet.

[7] Grover D, Mukerji M, Bhatnagar P, Kannan K, Samir K, Brahmachari SK (2004). Alu repeat analysis in the complete human genome: Trends and variations with respect to genomic composition. Bioinformatics.

[8] Mortada H, Vieira C, Lerat E (2010). Genes devoid of full-length transposable element insertions are involved in development and in the regulation of transcription in human and closely related species. J Mol Evol.

[9] Kidwell MG, Lisch DR (2000). Transposable elements and host genome evolution. Trends Ecol Evol.

[10] Biémont C, Vieira C (2006). Genetics: junk DNA as an evolutionary force. Nature.

[11] Kines KJ, Belancio VP (2012). Expressing genes do not forget their LINEs: transposable elements and gene expression. Front Biosci.

[12] Lynch VJ, Leclerc RD, May G, Wagner GP (2011). Transposon-mediated rewiring of gene regulatory networks contributed to the evolution of pregnancy in mammals. Nat Genet.

[13] Kunarso G, Chia N-Y, Jeyakani J, Hwang C, Lu X, Chan Y-S, Ng H-H, Bourque G (2010). Transposable elements have rewired the core regulatory network of human embryonic stem cells. Nat Genet.

[14] Lerat E, Sémon M (2007). Influence of the transposable element neighborhood on human gene expression in normal and tumor tissues. Gene.

[15] Belancio VP, Hedges DJ, Deininger P (2008). Mammalian non-LTR retrotransposons: For better or worse in sickness and in health. Genome Res.

[16] Hancks DC, Kazazian HH (2012). Active human retrotransposons: Variation and disease. Curr Opin Genet Dev.

[17] Straussman R, Nejman D, Roberts D, Steinfeld I, Blum B, Benvenisty N, Simon I, Yakhini Z, Cedar H (2009). Developmental programming of CpG island methylation profiles in the human genome. Nat Struct Mol Biol.

[18] Varley KE, Gertz J, Bowling KM, Parker SL, Reddy TE, Pauli-Behn F, Cross MK, Williams B, Stamatoyannopoulos J, Crawford GE (2013). Dynamic DNA methylation across diverse human cell lines and tissues. Genome Res.

[19] Ha M, Ng DW-K, Li W-H, Chen ZJ (2011). Coordinated histone modifications are associated with gene expression variation within and between species. Genome Res.

[20] Ghosh S, Yates AJ, Frühwald MC, Miecznikowski JC, Plass C, Smiraglia D (2010). Tissue specific DNA methylation of CpG islands in normal human adult somatic tissues distinguishes neural from non-neural tissues. Epigenetics.

[21] Kucharski R, Maleszka J, Foret S, Maleszka R (2008). Nutritional control of reproductive status in honeybees via DNA methylation. Science.

[22] Bird A (2002). DNA methylation patterns and epigenetic memory. Genes Dev.

[23] Bernstein BE, Meissner A, Lander ES (2007). The mammalian epigenome. Cell.

[24] Weber M, Schübeler D (2007). Genomic patterns of DNA methylation: targets and function of an epigenetic mark. Curr Opin Cell Biol.

[25] Jones PA, Liang G (2009). Rethinking how DNA methylation patterns are maintained. Nat Rev Genet.

[26] Carthew RW, Sontheimer EJ (2009). Origins and Mechanisms of miRNAs and siRNAs. Cell.

[27] Ghildiyal M, Zamore PD (2009). Small silencing RNAs: an expanding universe. Nat Rev Genet.

[28] Grant PA (2001). A tale of histone modifications. Genome Biol.

[29] Peterson CL, Laniel M-A (2004). Histones and histone modifications. Curr Biol.

[30] Li B, Carey M, Workman JL (2007). The role of chromatin during transcription. Cell.

[31] Vanyushin BF (2006). DNA methylation in plants. Curr Top Microbiol Immunol.

[32] Sharma S, Kelly TK, Jones P (2009). Epigenetics in cancer. Carcinogenesis.

[33] McKenna ES, Roberts CWM (2009). Epigenetics and cancer without genomic instability. Cell Cycle.

[34] Feinberg AP, Tycko B (2004). The history of cancer epigenetics. Nat Rev Cancer.

[35] Esteller M (2007). Cancer epigenomics: DNA methylomes and histone-modification maps. Nat Rev Genet.

[36] Slotkin RK, Martienssen R (2007). Transposable elements and the epigenetic regulation of the genome. Nat Rev Genet.

[37] Huda A, Jordan IK (2009). Epigenetic regulation of mammalian genomes by transposable elements. Ann N Y Acad Sci.

[38] Kulis M, Esteller M (2010). DNA methylation and cancer. Adv Genet.

[39] Ross JP, Rand KN, Molloy PL (2010). Hypomethylation of repeated DNA sequences in cancer. Epigenomics.

[40] Muster T, Waltenberger A, Grassauer A, Hirschl S, Caucig P, Romirer I, Seppele H, Schanab O, Magin-lachmann C, Lo R (2003). An endogenous retrovirus derived from human melanoma cells. Cancer Res.

[41] Florl AR, Löwer R, Schmitz-Dräger BJ, Schulz WA (1999). DNA methylation and expression of LINE-1 and HERV-K provirus sequences in urothelial and renal cell carcinomas. Br J Cancer.

[42] Smith IM, Mydlarz WK, Mithani SK, Califano JA (2007). DNA global hypomethylation in squamous cell head and neck cancer associated with smoking alcohol consumption and stage. Int J Cancer.

[43] Depil S, Roche C, Dussart P, Prin L (2002). Expression of a human endogenous retrovirus HERV-K in the blood cells of leukemia patients. Leukemia.

[44] Patzke S, Lindeskog M, Munthe E, Aasheim HC (2002). Characterization of a novel human endogenous retrovirus HERV-H/F expressed in human leukemia cell lines. Virology.

[45] Debniak T, Gorski B, Cybulski C, Jakubowska A, Kurzawski G, Kladny J, Lubinski J (2001). Comparison of Alu-PCR microsatelite instability and immunohistochemical analyses in finding features characteristic for hereditary nonpolyposis colorectal cancer. J Cancer Res Clin Oncol.

[46] Wang-Johanning F, Liu J, Rycaj K, Huang M, Tsai K, Rosen DG, Chen D-T, Lu DW, Barnhart KF, Johanning GL (2007). Expression of multiple human endogenous retrovirus surface envelope proteins in ovarian cancer. Int J Cancer.

[47] Wang-Johanning F, Frost AR, Johanning GL, Khazaeli MB, LoBuglio AF, Shaw DR, Strong TV (2001). Expression of human endogenous retrovirus k envelope transcripts in human breast cancer. Clin Cancer Res.

[48] Menendez L, Benigno BB, McDonald JF (2004). L1 and HERV-W retrotransposons are hypomethylated in human ovarian carcinomas. Mol Cancer.

[49] Chénais B (2013). Transposable elements and human cancer: A causal relationship?. Biochim Biophys Acta.

[50] Fraga MF, Ballestar E, Villar-Garea A, Boix-Chornet M, Espada J, Schotta G, Bonaldi T, Haydon C, Ropero S, Petrie K (2005). Loss of acetylation at Lys16 and trimethylation at Lys20 of histone H4 is a common hallmark of human cancer. Nat Genet.

[51] Gendrel A-V, Lippman Z, Yordan C, Colot V, Martienssen RA (2002). Dependence of heterochromatic histone H3 methylation patterns on the Arabidopsis gene DDM1. Science.

[52] Volpe TA, Kidner C, Hall IM, Teng G, Grewal SIS, Martienssen RA (2002). Regulation of heterochromatic silencing and histone H3 lysine-9 methylation by RNAi. Science.

[53] Lippman Z, Gendrel A-V, Black M, Vaughn MW, Dedhia N, McCombie WR, Lavine K, Mittal V, May B, Kasschau KD (2004). Role of transposable elements in heterochromatin and epigenetic control. Nature.

[54] Rebollo R, Karimi MM, Bilenky M, Gagnier L, Miceli-Royer K, Zhang Y, Goyal P, Keane TM, Jones S, Hirst M (2011). Retrotransposon-induced heterochromatin spreading in the mouse revealed by insertional polymorphisms. PLoS Genet.

[55] Smedley D, Haider S, Durinck S, Pandini L, Provero P, Allen J, Arnaiz O, Awedh MH, Baldock R, Barbiera G (2015). The BioMart community portal: an innovative alternative to large centralized data repositories. Nucleic Acids Res.

[56] Smit AFA, Hubley R, Green P. RepeatMasker Open-30. 1996–2010. http://www.repeatmasker.org

[57] Jurka J, Kapitonov VV, Pavlicek A, Klonowski P, Kohany O, Walichiewicz J (2005). Repbase Update a database of eukaryotic repetitive elements. Cytogenet Genome Res.

[58] Bailly-Bechet M, Haudry A, Lerat E (2014). ‘One code to find them all’: a perl tool to conveniently parse RepeatMasker output files. Mob DNA.

[59] ENCODE Project Consortium (2012). An integrated encyclopedia of DNA elements in the human genome. Nature.

[60] Barski A, Cuddapah S, Cui K, Roh T-Y, Schones DE, Wang Z, Wei G, Chepelev I, Zhao K (2007). High-resolution profiling of histone methylations in the human genome. Cell.

[61] Barth TK, Imhof A (2010). Fast signals and slow marks: the dynamics of histone modifications. Trends Biochem Sci.

[62] R core team. https://www.r-project.org/. 2015.

[63] Han J Kamber M, Pei J. Data Mining: concepts and techniques. Elsevier. 2012. Morgan Kaufmann Publishers, July 2011. ISBN 978-0123814791

[64] Modolo L, Lerat E (2015). UrQt: an efficient software for the Unsupervised Quality trimming of NGS data. BMC Bioinformatics.

[65] Kim D, Pertea G, Trapnell C, Pimentel H, Kelley R, Salzberg SL (2013). TopHat2: accurate alignment of transcriptomes in the presence of insertions deletions and gene fusions. Genome Biol.

[66] Anders S, Pyl PT, Huber W (2014). HTSeq - A Python framework to work with high-throughput sequencing data. Bioinformatics.

[67] Love MI, Huber W, Anders S (2014). Moderated estimation of fold change and dispersion for RNA-seq data with DESeq2. Genome Biol.

[68] Eden E, Navon R, Steinfeld I, Lipson D, Yakhini Z (2009). GOrilla: a tool for discovery and visualization of enriched GO terms in ranked gene lists. BMC Bioinformatics.

[69] Supek F, Bošnjak M, Škunca N, Šmuc T (2011). REVIGO summarizes and visualizes long lists of gene ontology terms. PLoS One.

[70] Montoya-Durango DE, Liu Y, Teneng I, Kalbfleisch T, Lacy ME, Steffen MC, Ramos KS (2009). Epigenetic control of mammalian LINE-1 retrotransposon by retinoblastoma proteins. Mutat Res.

[71] Rangasamy D (2013). Distinctive patterns of epigenetic marks are associated with promoter regions of mouse LINE-1 and LTR retrotransposons. Mob DNA.

[72] Forbes SA, Beare D, Gunasekaran P, Leung K, Bindal N, Boutselakis H, Ding M, Bamford S, Cole C, Ward S (2014). COSMIC: exploring the world’s knowledge of somatic mutations in human cancer. Nuc Acids Res.

[73] Huisinga KL, Brower-Toland B, Elgin SCR (2006). The contradictory definitions of heterochromatin: transcription and silencing. Chromosoma.

[74] Koch CM, Andrews RM, Flicek P, Dillon SC, Karaöz U, Clelland GK, Wilcox S, Beare DM, Fowler JC, Couttet P (2007). The landscape of histone modifications across 1 % of the human genome in five human cell lines. Genome Res.

[75] Karlić R, Chung H-R, Lasserre J, Vlahovicek K, Vingron M (2010). Histone modification levels are predictive for gene expression. Proc Natl Acad Sci U S A.

[76] Kvikstad EM, Makova KD (2010). The (r)evolution of SINE versus LINE distributions in primate genomes: Sex chromosomes are important. Genome Res.

[77] Woo YH, Li W-H (2012). Evolutionary conservation of histone modifications in mammals. Mol Biol Evol.

[78] Kondo Y, Issa J-PJ (2003). Enrichment for histone H3 lysine 9 methylation at Alu repeats in human cells. J Biol Chem.

[79] Martens JH, O’Sullivan RJ, Braunschweig U, Opravil S, Radolf M, Steinlein P, Jenuwein T (2005). The profile of repeat-associated histone lysine methylation states in the mouse epigenome. EMBO J.

[80] Pauler FM, Sloane MA, Huang R, Regha K, Koerner MV, Tamir I, Sommer A, Aszodi A, Jenuwein T, Barlow DP (2009). H3K27me3 forms BLOCs over silent genes and intergenic regions and specifies a histone banding pattern on a mouse autosomal chromosome. Genome Res.

[81] Mikkelsen TS, Ku M, Jaffe DB, Issac B, Lieberman E, Giannoukos G, Alvarez P, Brockman W, Kim T-K, Koche RP (2007). Genome-wide maps of chromatin state in pluripotent and lineage-committed cells. Nature.

[82] Huda A, Bowen NJ, Conley AB, Jordan IK (2011). Epigenetic regulation of transposable element derived human gene promoters. Gene.

[83] Eichten SR, Ellis NA, Makarevitch I, Yeh CT, Gent JI, Guo L, McGinnis KM, Zhang X, Schnable PS, Vaughn MW (2012). Spreading of Heterochromatin Is Limited to Specific Families of Maize Retrotransposons. PLoS Genet.

[84] Cheung P, Tanner KG, Cheung WL, Sassone-Corsi P, Denu JM, Allis CD (2000). Synergistic coupling of histone H3 phosphorylation and acetylation in response to epidermal growth factor stimulation. Mol Cell.

[85] Füllgrabe J, Kavanagh E, Joseph B (2011). Histone onco-modifications. Oncogene.

[86] Portela A, Esteller M (2010). Epigenetic modifications and human disease. Nat Biotechnol.

[87] Huda A, Mariño-Ramírez L, Jordan IK (2010). Epigenetic histone modifications of human transposable elements: genome defense versus exaptation. Mob DNA.

[88] Bert SA, Robinson MD, Strbenac D, Statham AL, Song JZ, Hulf T, Sutherland RL, Coolen MW, Stirzaker C, Clark SJ (2013). Regional activation of the cancer genome by long-range epigenetic remodeling. Cancer Cell.

[89] Dudziec E, Gogol-Döring A, Cookson V, Chen W, Catto J (2013). Integrated epigenome profiling of repressive histone modifications DNA methylation and gene expression in normal and malignant urothelial cells. PLoS One.

[90] Aporntewan C, Phokaew C, Piriyapongsa J, Ngamphiw C, Ittiwut C, Tongsima S, Mutirangura A (2011). Hypomethylation of intragenic LINE-1 represses transcription in cancer cells through AGO2. PLoS One.

[91] Abrusán G (2012). Somatic transposition in the brain has the potential to influence the biosynthesis of metabolites involved in Parkinson’s disease and schizophrenia. Biol Direct.

[92] Lee E, Iskow R, Yang L, Gokcumen O, Haseley P, Luquette LJ, Lohr JG, Harris CC, Ding L, Wilson RK (2012). Landscape of somatic retrotransposition in human cancers. Science.

[93] Solyom S, Ewing AD, Rahrmann EP, Doucet T, Nelson HH, Burns MB, Harris RS, Sigmon DF, Casella A, Erlanger B (2012). Extensive somatic L1 retrotransposition in colorectal tumors. Genome Res.

[94] Helman E, Lawrence MS, Stewart C, Sougnez C, Getz G, Meyerson M (2014). Somatic retrotransposition in human cancer revealed by whole-genome and exome sequencing. Genome Res.

[95] Ewing AD, Gacita A, Wood LD, Ma F, Xing D, Kim M-S, Manda SS, Abril G, Pereira G, Makohon-Moore A (2015). Widespread somatic L1 retrotransposition occurs early during gastrointestinal cancer evolution. Genome Res.

[96] Prendergast JGD, Chambers EV, Semple AM (2014). Sequence-level mechanisms of human epigenome evolution. Genome Biol Evol.

[97] Rishishwar L, Tellez Villa CE, Jordan IK (2015). Transposable element polymorphisms recapitulate human evolution. Mob DNA.

