# Supplementary material for: The transposable element environment of human genes is associated with histone and expression changes in cancer
Source: BMC Genomics. 2016 Aug 9;17:588. doi: 10.1186/s12864-016-2970-1 (PMC4979156; doi:10.1186/s12864-016-2970-1)
Supplement: Additional file 1: Figure S2. — Distribution of the TE density and TE coverage of genes along all chromosomes. (PDF 311 kb) [file 12864_2016_2970_MOESM1_ESM.pdf]

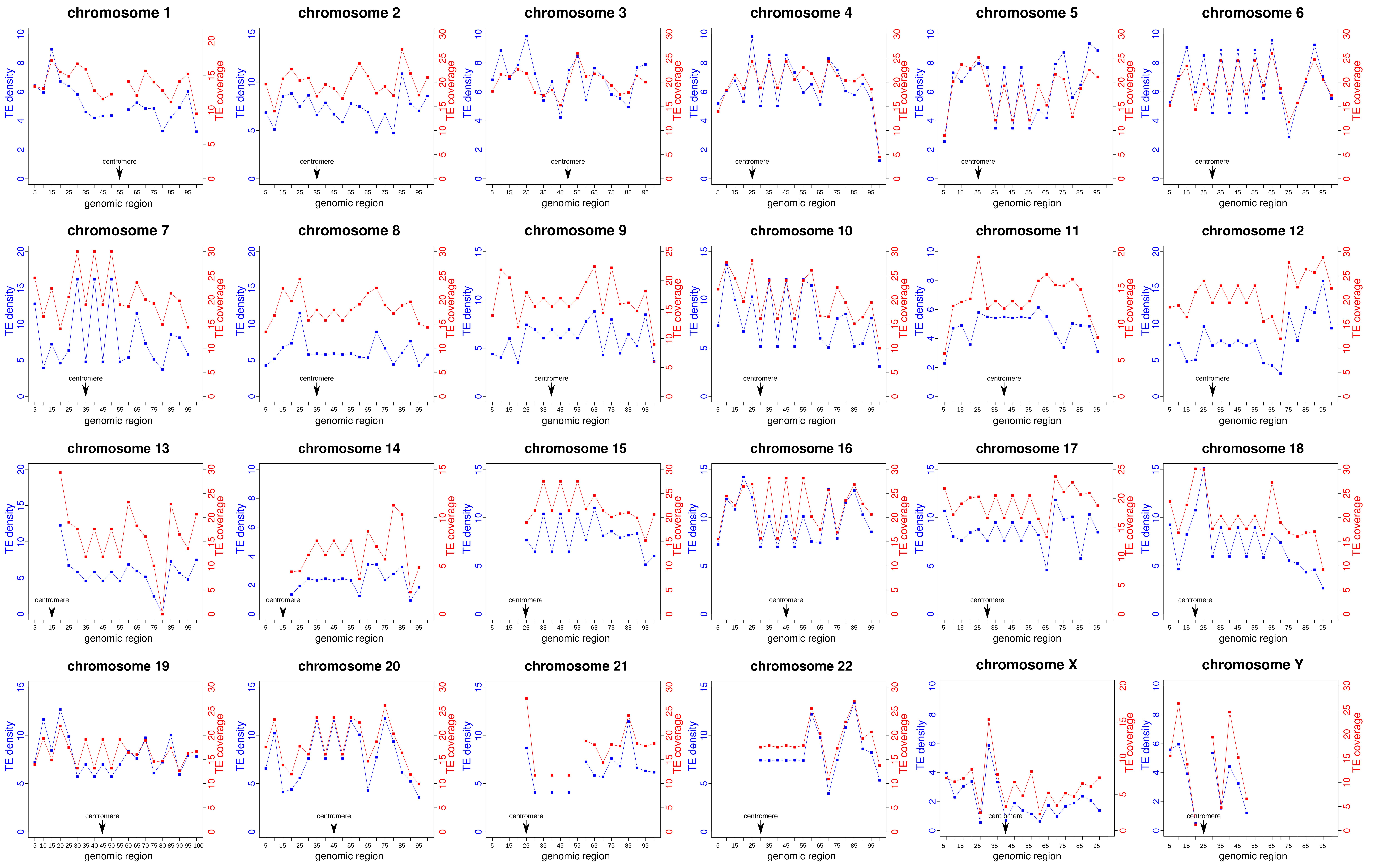
chromosome 1
chromosome 2
chromosome 3
chromosome 4
chromosome 5
chromosome 6
chromosome 7
chromosome 8
chromosome 9
chromosome 10
chromosome 11
chromosome 12
chromosome 13
chromosome 14
chromosome 15
chromosome 16
chromosome 17
chromosome 18
chromosome 19
chromosome 20
chromosome 21
chromosome 22
chromosome X
chromosome Y
TE density
TE coverage
genomic region
centromere
