# Supplementary material for: The transposable element environment of human genes is associated with histone and expression changes in cancer
Source: BMC Genomics. 2016 Aug 9;17:588. doi: 10.1186/s12864-016-2970-1 (PMC4979156; doi:10.1186/s12864-016-2970-1)
Supplement: Additional file 2: Table S1. — Mean enrichment of genes responsible for the high mean enrichment value for H3K27ac and/or H3K4me3 at particular positions of sex chromosomes. (PDF 207 kb) [file 12864_2016_2970_MOESM2_ESM.pdf]

Table S1: Mean enrichment of genes responsible for the high mean enrichment value for H3K27ac and/or H3K4me3 at particular positions of sex chromosomes

chromosome	start	genes	**h3k27ac**	h3k27me3	h3k36me3	h3k4me1	h3k4me2	**h3k4me3**	h3k79me2	h3k9ac	h3k9me3	h4k20me1	bin
X	15645441	ENSG00000147003	**97.63**	2.98	0	6.52	0	0	11.94	0	0	0	
X	15706953	ENSG00000169239	**123.42**	15.32	39.74	37.78	91.76	**50.32**	35.57	62.82	7.20	0	
X	16606126	ENSG00000047230	4.31	8.85	49.64	1.13	3.63	**49.89**	3.27	2.08	0.51	0.33	15
X	17393543	ENSG00000188158	**887.27**	1.38	0	24.54	61.10	34.50	10.43	27.85	0.30	7.51	
X	17755588	ENSG00000047634	**123.57**	2.31	1.84	1.96	26.87	7.93	4.98	0	0	0	
X	23720370	ENSG00000123130	0.08	0	0.06	62.47	151.28	**79.03**	147.48	117.79	30.17	0	
X	24072833	ENSG00000130741	**124.86**	4.63	14.54	18.47	55.07	38.70	25.54	103.54	0	7.12	20
X	27826107	ENSG00000177689	**450.15**	18.05	0	0	1.45	29.04	0	3.36	0	0	
X	40488285	ENSG00000185753	**89.10**	0	6.35	168.87	85.51	**57.44**	23.57	94.67	0	15.35	
X	41192651	ENSG00000215301	**570.79**	0	0.81	1.18	1.60	**118.32**	76.35	3.14	0	0.39	30
X	44007128	ENSG00000183690	**8555.18**	0.77	0	3.57	172.96	**1018.59**	0	1.23	0.28	0	
X	73805052	ENSG00000131263	**73.92**	1.02	24.71	15.92	7.92	6.36	19.20	57.09	13.67	1.94	
X	150732094	ENSG00000166049	**11234.56**	3.44	0	0	0	**272.89**	0	113.14	0	0.21	
X	152965947	ENSG00000185825	**52.41**	0	54.94	0.18	0.38	0.25	69.41	43.95	0.05	0.17	
X	153126969	ENSG00000198910	**190.82**	11.63	14.82	39.85	55.88	32.97	0	22.41	4.99	9.29	50
X	153618315	ENSG00000147403	8.67	0	1.10	3.07	68.48	**44.16**	4.39	4.21	1.03	1.12	
X	153686621	ENSG00000130827	**54.86**	0	8.97	2.82	3.25	29.75	27.20	53.55	6.79	0	
X	153706028	ENSG00000196976	**58.90**	0	0	16.05	42.94	21.68	22.14	33.02	0	0	

In bold are highlighted values above the mean enrichment for the considered histone modification
