# Supplementary material for: The transposable element environment of human genes is associated with histone and expression changes in cancer
Source: BMC Genomics. 2016 Aug 9;17:588. doi: 10.1186/s12864-016-2970-1 (PMC4979156; doi:10.1186/s12864-016-2970-1)
Supplement: Additional file 3: Table S2. — Mean enrichment of genes responsible for the high mean enrichment value for H3K27ac H3K4me3 and/or H3K9ac at particular positions of autosomal chromosomes. (PDF 260 kb) [file 12864_2016_2970_MOESM3_ESM.pdf]

Table S2: Mean enrichment of genes responsible for the high mean enrichment value for H3K27ac, H3K4me3 and/or H3K9ac at particular positions of autosomal chromosomes

chromosome	start	gene	**h3k27ac**	h3k27me3	h3k36me3	h3k4me1	h3k4me2	**h3k4me3**	h3k79me2	**h3k9ac**	h3k9me3	h4k20me1	bin
1	50905150	ENSG00000185104	**58.76**	0.50	1.44	34.09	59.72	**30.56**	0.66	**49.64**	0.98	0.44	25
	53971910	ENSG00000174332	**306.20**	7.07	0	19.04	0	**31.26**	0	0	222.22	1.81	
	55532032	ENSG00000162402	**167.70**	35.16	0	142.66	255.43	**154.16**	2.25	**298.24**	0.55	0.59	
	59041099	ENSG00000184292	**80.09**	0	0	11.92	0.23	9.19	4.62	**56.00**	6.86	0	
2	50145643	ENSG00000179915	3.34	0.28	0	2.44	11.81	3.02	0	**59.87**	0	0	
	55774428	ENSG00000138041	**580.10**	0	0.76	1.37	195.94	**305.12**	0.67	**618.96**	0.38	0.40	
	58386378	ENSG00000115392	1.43	0	0.63	54.61	79.15	**75.81**	0.58	**145.91**	0.27	0	
3	42055294	ENSG00000182606	2.22	0	2.00	0.82	1.55	1.07	1.43	**63.07**	0.78	0.09	
	42850975	ENSG00000240747	**66.01**	0.46	0	4.33	10.29	14.71	0	0	4.11	0	
	48725436	ENSG00000068745	**45.59**	1.78	0.50	40.37	56.30	**35.41**	0.83	**102.43**	0	16.16	
	49315264	ENSG00000114316	0.23	0	0	97.01	0.20	0.10	0	**158.06**	0.10	0.02	
4	39046659	ENSG00000109790	0.92	0	0.23	0.23	0.52	0.13	0	**72.10**	0.04	0	
	39184024	ENSG00000157796	**96.38**	0	10.55	6.25	17.93	7.62	0	44.24	0	7.65	
	46250444	ENSG00000151834	**87.71**	20.34	0	1.03	0	14.90	0	0	0	0	
5	41925356	ENSG00000151876	**55.47**	0	0	4.84	12.54	9.25	1.84	0.98	0	0	
6	35182190	ENSG00000146197	**177.31**	0.36	1.85	5.04	8.45	15.86	3.23	6.50	3.58	1.88	
	35420138	ENSG00000112039	7.51	18.91	135.83	2.85	350.16	**321.60**	479.13	**491.02**	0	8.98	
	41158015	ENSG00000112195	**46.90**	0.09	0.13	34.36	53.07	2.04	0.25	39.45	0.40	0.11	
7	39605975	ENSG00000241127	1.58	8.43	2.84	94.38	1.47	1.27	4.21	**191.72**	0	0	
	39663082	ENSG00000006451	35.37	0.19	34.90	12.50	50.96	21.38	22.59	**71.38**	1.06	25.33	
8	29920528	ENSG00000133872	**53.93**	0	1.23	0.19	39.18	0.14	29.57	**61.37**	0.30	0.26	
	33405273	ENSG00000133874	**46.41**	0	30.97	38.52	55.35	**39.56**	31.26	**50.34**	0	0.16	
	35092975	ENSG00000156687	**193.58**	0	0	0.26	0	0.33	0	0	0.47	0	
9	32540542	ENSG00000197579	**50.51**	0	1.29	9.86	1.77	18.53	1.34	28.30	0	0	
	33247818	ENSG00000107262	**159.93**	0	0	5.03	49.21	4.60	0	**89.77**	46.73	0	

chromosome	start	gene	**h3k27ac**	h3k27me3	h3k36me3	h3k4me1	h3k4me2	**h3k4me3**	h3k79me2	**h3k9ac**	h3k9me3	h4k20me1	bin
10	32297938	ENSG00000170759	**99.04**	0	25.36	38.72	41.69	**57.53**	49.86	**92.08**	3.29	6.53	
11	27676440	ENSG00000176697	**95.68**	0.30	0	0.41	1.48	1.158	0	1.36	4.34	2.73	
	31833939	ENSG00000049449	**356.47**	0.22	14.28	251.97	453.45	**248.80**	17.42	**453.35**	0.57	4.77	
12	29490285	ENSG00000087502	43.13	0	15.38	21.66	9.11	**37.00**	12.62	24.43	23.70	0	
13	25875662	ENSG00000139496	**73.60**	5.11	0.54	0.85	1.89	1.27	3.38	3.79	18.28	6.18	
14	21558205	ENSG00000165804	**85.50**	0	0	2.89	6.19	4.55	0.67	8.54	0	0	
	23235731	ENSG00000155463	**479.92**	0	0.80	1.73	257.12	**267.17**	1.7038	**592.44**	0	0.02	
	24549316	ENSG00000129535	**113.72**	0	28.25	0.63	1.17	0.93	0.82	1.89	0.56	0	
	24600484	ENSG00000139914	**77.92**	0	0	18.23	71.62	**70.72**	1.09	**90.27**	0	0	
	24658349	**ENSG00000100926**	**30736.60**	0	1.23	2.28	3.77	**19023.78**	2.92	**36315.75**	2.86	0	
	24683161	ENSG00000255526	**393.04**	0	2.11	112.11	11.18	**328.27**	164.63	**386.67**	0	2.75	
	24774302	ENSG00000136305	**113.50**	0.17	0	0	112.79	**47.16**	0	**95.96**	0	0	
	24895738	ENSG00000139899	**100.03**	0.78	24.08	31.30	5.23	3.72	39.76	**98.48**	0	0	
16	21963981	ENSG00000140740	44.06	2.73	4.78	12.30	24.08	28.71	8.23	25.03	0	1.49	
	22018959	ENSG00000185716	**48.15**	0	0.289	26.51	49.04	23.48	6.66	**49.03**	1.07	1.02	
	22308730	ENSG00000058600	2.88	0	0	14.84	24.64	25.37	0.71	**70.44**	7.55	0.95	
19	11998599	ENSG00000198429	3.21	0	0	2.53	3.20	2.38	0	**50.31**	1.84	0	
20	13246709	ENSG00000089123	**51.46**	14.93	7.47	13.24	32.36	16.01	15.24	28.73	1.27	0	
1	148555979	ENSG00000243452	4.60	0	1.29	41.91	111.14	**65.70**	1.94	**134.20**	0	0	60
6	99282580	ENSG00000184486	7.94	0.76	0	145.41	0.79	**307.21**	0	0	0.27	0.16	
9	77675489	ENSG00000106733	11.90	0	0.49	0.65	1.18	**67.13**	3.92	6.11	11.76	1.79	
	79000433	ENSG00000135002	26.10	0.17	6.55	113.13	182.52	**151.39**	17.91	28.42	0.29	0	
10	78629359	ENSG00000156113	11.58	5.43	0	59.12	58.89	**64.91**	0	0.843	0.15	0.39	
12	77157368	ENSG00000186908	11.75	1.25	7.60	32.15	87.61	**45.11**	32.15	**106.87**	6.90	0.68	
14	59100685	ENSG00000165617	**102.90**	0.35	0	2.50	261.95	**130.02**	0	43.57	0	0	
	60062694	**ENSG00000139970**	**231.61**	1.12	0	3357.63	6.01	**25738.94**	0	0	2.40	6.93	
22	29950797	ENSG00000184117	**737.68**	0	1.35	119.17	1150.70	**550.23**	449.96	**575.70**	0	0	

chromosome	start	gene	**h3k27ac**	h3k27me3	h3k36me3	h3k4me1	h3k4me2	**h3k4me3**	h3k79me2	**h3k9ac**	h3k9me3	h4k20me1	bin
	30126945	ENSG00000100319	**276.60**	0	36.59	26.57	213.20	**83.01**	5.65	**417.31**	5.19	1.15	
1	179851177	ENSG00000143337	**85.04**	0	0	15.46	0	**32.79**	0	**60.34**	0	0	75
2	173940163	ENSG00000091436	**176.05**	0.07	1.48	0.90	1.59	0.89	1.01	1.54	0.16	0.45	
7	112459202	ENSG00000164603	**50.85**	0.35	0.31	5.32	15.37	7.18	0.45	**47.99**	0.62	0	
	113516832	ENSG00000154415	**61.07**	5.09	0	32.45	19.85	**32.82**	0	0	0	0	
8	103216730	ENSG00000048392	**65.86**	0	12.46	17.18	45.91	29.34	35.65	**47.25**	1.53	0	
9	97021593	ENSG00000175787	**103.97**	0.58	4.14	23.97	43.75	29.55	42.40	**137.86**	29.30	0.24	
	99148223	ENSG00000165244	**81.74**	0	0.52	0.53	1.02	0.60	30.43	**62.91**	0.14	0	
	100615536	ENSG00000178919	**45.24**	5.97	0	0.19	0.45	0.28	0	0	3.27	2.79	
	101050391	ENSG00000136928	**126.49**	82.89	0	2.43	6.87	1.40	0	1.83	100.45	0.91	
	102668915	ENSG00000136874	**99.86**	0	0.37	0.18	0.40	0.24	0.16	**206.76**	0.68	0	
10	97951458	ENSG00000095585	**69.69**	0	0.25	40.38	0.33	0.20	0.45	**63.84**	0.49	0.32	
	99092255	ENSG00000181274	**53.36**	10.36	0	8.16	17.64	11.98	14.95	17.03	0	8.80	
11	95709762	ENSG00000184384	**56.27**	0	19.21	5.68	5.86	26.52	10.09	42.94	0.06	1.87	
	96123153	ENSG00000183340	**84.75**	0.68	0	1.82	0	7.42	0	0	34.34	0	
14	75230069	**ENSG00000119596**	**7329.16**	0	7.81	53.72	26.81	19.58	3331.85	33.77	0.67	0.23	
	77253588	ENSG00000013523	**46.66**	1.88	57.10	47.91	85.77	**70.23**	38.01	**81.04**	0	0	
	77731826	ENSG00000165553	**735.90**	0.45	10.76	0	7.83	**88.60**	0	21.71	0	2.32	
15	74287014	ENSG00000140464	**123.90**	15.27	0	0.18	0.25	0.60	0	2.65	1.00	3.83	
16	67218269	ENSG00000179044	**69.04**	0.17	6.94	3.43	59.95	**58.87**	2.11	2.34	0	0	
	67241042	ENSG00000125122	**52.83**	0	0.54	32.65	92.31	**73.22**	2.93	4.06	0	0.38	
19	42387228	ENSG00000076928	**49.15**	1.95	3.55	1.53	6.79	1.81	0	2.43	0.77	11.05	
	42829761	ENSG00000105429	**254.18**	118.26	2.02	1.51	2.96	13.69	2.58	7.90	1.61	0.87	
20	45169585	ENSG00000149635	**99.67**	0.39	0	62.69	1.74	**46.45**	0	0.98	0.37	0	
21	33683329	ENSG00000142207	**737.38**	198.17	0	0.22	0.44	0.33	0.24	**634.62**	0.27	0	
	34696734	ENSG00000142166	**62.81**	5.23	0	33.87	44.48	25.37	34.27	**64.54**	23.27	1.73	

chromosome	start	gene	**h3k27ac**	h3k27me3	h3k36me3	h3k4me1	h3k4me2	**h3k4me3**	h3k79me2	**h3k9ac**	h3k9me3	h4k20me1	bin
22	36677327	ENSG00000100345	**161.59**	0	0	102.61	184.87	**131.44**	0.59	**206.05**	0.17	0.73	
	37521878	ENSG00000100385	**77.11**	1.03	1.13	47.77	56.71	**32.49**	7.17	**67.41**	301.97	8.39	
	38004481	ENSG00000100083	**171.01**	0.63	1.72	122.95	173.01	**111.46**	2.87	**184.46**	0	0.70	

In bold are highlighted values above the mean enrichment for the considered histone modification
