# Supplementary material for: The transposable element environment of human genes is associated with histone and expression changes in cancer
Source: BMC Genomics. 2016 Aug 9;17:588. doi: 10.1186/s12864-016-2970-1 (PMC4979156; doi:10.1186/s12864-016-2970-1)
Supplement: Additional file 4: Figure S1. — Distribution of the mean histone enrichment along all chromosomes for the 10 histone modifications in the normal condition (GM12878). The chromosome Y is not represented since all of its 45 genes did not present any histone enrichment values. (PDF 252 kb) [file 12864_2016_2970_MOESM4_ESM.pdf]

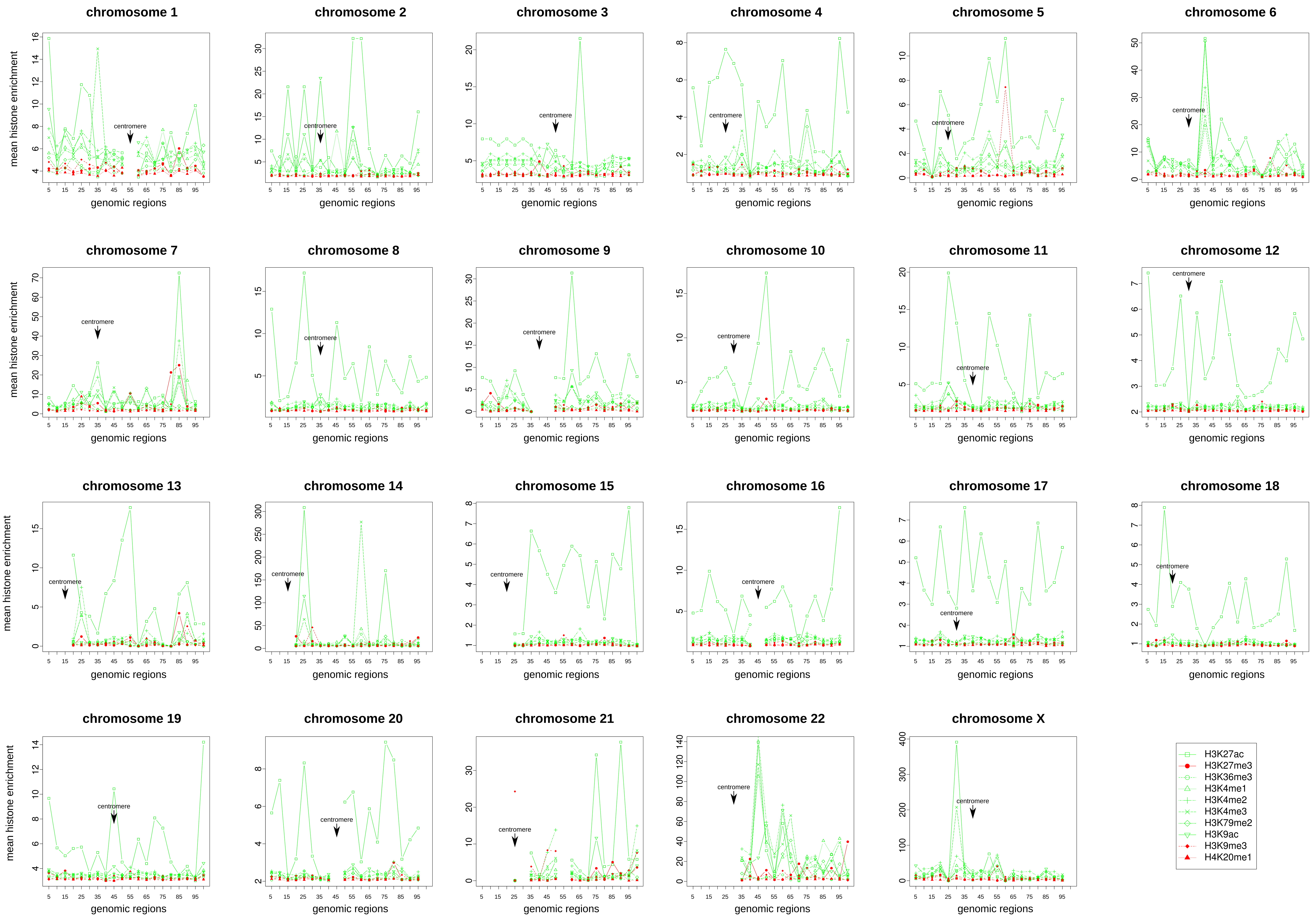
chromosome 1
chromosome 2
chromosome 3
chromosome 4
chromosome 5
chromosome 6
chromosome 7
chromosome 8
chromosome 9
chromosome 10
chromosome 11
chromosome 12
chromosome 13
chromosome 14
chromosome 15
chromosome 16
chromosome 17
chromosome 18
chromosome 19
chromosome 20
chromosome 21
chromosome 22
chromosome X
mean histone enrichment
genomic regions
centromere
H3K27ac
H3K27me3
H3K36me3
H3K4me1
H3K4me2
H3K4me3
H3K79me2
H3K9ac
H3K9me3
H4K20me1
