# Supplementary material for: The transposable element environment of human genes is associated with histone and expression changes in cancer
Source: BMC Genomics. 2016 Aug 9;17:588. doi: 10.1186/s12864-016-2970-1 (PMC4979156; doi:10.1186/s12864-016-2970-1)
Supplement: Additional file 5: Table S3. — Mean values of histone enrichment for each histone modification and for each gene category. (PDF 231 kb) [file 12864_2016_2970_MOESM5_ESM.pdf]

Table S3: Mean values of histone enrichment for each histone modification and for each gene category

	H3K27ac			wilcoxon non paired tests p-values		
	**TE_free**	**TE_intermediate**	**TE_rich**	free/rich	free/intermediate	intermediate/rich
**normal**	11.69	7.128	14.27	< 2.2e-16	< 2.2e-16	< 2.2e-16
**cancer**	4.216	5.66	10.13	< 2.2e-16	< 2.2e-16	< 2.2e-16
wilcoxon paired tests p-value	0.4565 (NS)	1.66e-009	0.3216 (NS)			
	H3K27me3			wilcoxon non paired tests p-values		
	**TE_free**	**TE_intermediate**	**TE_rich**	free/rich	free/intermediate	intermediate/rich
**normal**	3.136	2.19	1.871	1.10e-012	< 2.2e-16	< 2.2e-16
**cancer**	6.116	3.307	4.132	< 2.2e-16	< 2.2e-16	8.70E-010
wilcoxon paired tests p-value	7.23e-006	< 2.2e-16	< 2.2e-16			
	H3K36me3			wilcoxon non paired tests p-values		
	**TE_free**	**TE_intermediate**	**TE_rich**	free/rich	free/intermediate	intermediate/rich
**normal**	2.202	1.624	2.457	< 2.2e-16	< 2.2e-16	< 2.2e-16
**cancer**	1.722	1.936	3.958	< 2.2e-16	< 2.2e-16	< 2.2e-16
wilcoxon paired tests p-values	< 2.2e-16	< 2.2e-16	< 2.2e-16			
	H3K4me1			wilcoxon non paired tests p-values		
	**TE_free**	**TE_intermediate**	**TE_rich**	free/rich	free/intermediate	intermediate/rich
**normal**	5.425	4.786	6.922	< 2.2e-16	< 2.2e-16	< 2.2e-16
**cancer**	7.881	4.679	6.917	< 2.2e-16	< 2.2e-16	< 2.2e-16
wilcoxon paired tests p-values	4.89e-005	2.85e-008	< 2.2e-16			
	H3K4me2			wilcoxon non paired tests p-values		
	**TE_free**	**TE_intermediate**	**TE_rich**	free/rich	free/intermediate	intermediate/rich
**normal**	6.8	6.446	9.148	< 2.2e-16	< 2.2e-16	< 2.2e-16
**cancer**	6.737	8.469	12.72	< 2.2e-16	< 2.2e-16	< 2.2e-16
wilcoxon paired tests p-values	< 2.2e-16	< 2.2e-16	< 2.2e-16			
	H3K79me2			wilcoxon non paired tests p-values		
	**TE_free**	**TE_intermediate**	**TE_rich**	free/rich	free/intermediate	intermediate/rich
**normal**	2.715	2.392	4.477	< 2.2e-16	< 2.2e-16	< 2.2e-16

**cancer**	1.876	2.666	3.662	< 2.2e-16	< 2.2e-16	< 2.2e-16
wilcoxon paired tests p-values	0.2783 (NS)	0.007749 (NS)	0.03283 (NS)			
	**H3K9ac**			wilcoxon non paired tests p-values		
	**TE_free**	**TE_intermediate**	**TE_rich**	free/rich	free/intermediate	intermediate/rich
**normal**	6.007	5.173	15.49	< 2.2e-16	< 2.2e-16	< 2.2e-16
**cancer**	3.567	4.724	7.983	< 2.2e-16	< 2.2e-16	< 2.2e-16
wilcoxon paired tests p-values	0.007121 (NS)	< 2.2e-16	< 2.2e-16			
	**H3K9me3**			wilcoxon non paired tests p-values		
	**TE_free**	**TE_intermediate**	**TE_rich**	free/rich	free/intermediate	intermediate/rich
**normal**	2.139	1.638	1.953	< 2.2e-16	< 2.2e-16	< 2.2e-16
**cancer**	0.8863	1.215	2.204	< 2.2e-16	< 2.2e-16	< 2.2e-16
wilcoxon paired tests p-values	< 2.2e-16	< 2.2e-16	< 2.2e-16			
	**H4K20me1**			wilcoxon non paired tests p-values		
	**TE_free**	**TE_intermediate**	**TE_rich**	free/rich	free/intermediate	intermediate/rich
**normal**	0.7087	0.9031	1.116	< 2.2e-16	< 2.2e-16	< 2.2e-16
**cancer**	2.025	1.719	2.268	< 2.2e-16	< 2.2e-16	< 2.2e-16
wilcoxon paired tests p-values	< 2.2e-16	< 2.2e-16	< 2.2e-16			
	**H3K4me3**			wilcoxon non paired tests p-values		
	**TE_free**	**TE_intermediate**	**TE_rich**	free/rich	free/intermediate	middle/rich
**normal**	11.59	4.907	10.82	< 2.2e-16	< 2.2e-16	< 2.2e-16
**cancer**	4.227	6.538	8.861	< 2.2e-16	< 2.2e-16	< 2.2e-16
wilcoxon paired tests p-values	< 2.2e-16	< 2.2e-16	< 2.2e-16			
