# Supplementary material for: The transposable element environment of human genes is associated with histone and expression changes in cancer
Source: BMC Genomics. 2016 Aug 9;17:588. doi: 10.1186/s12864-016-2970-1 (PMC4979156; doi:10.1186/s12864-016-2970-1)
Supplement: Additional file 6: Table S4. — Differential mean values of enrichment for each histone modification and for each gene category according to the TE type. (PDF 31 kb) [file 12864_2016_2970_MOESM6_ESM.pdf]

Table S4: Differential mean values of enrichment for each histone modification and for each gene category according to the TE type.

	Differential mean enrichment (Normal - Cancer)									
	H3K27ac	H3K9ac	H3K36me3	H3K4me1	H3K4me2	H3K4me3	H3K79me2	H3K27me3	H3K9me3	H4K20me1
**SINE_rich**	10.73	14.58	-2.21	0.48	-8.26	5.57	0.58	-0.24	-2.44	-0.91
**LINE_rich**	0.65	-0.13	-0.48	0.96	-0.89	-3.48	-0.29	-1.19	0.51	-0.43
**LTR_rich**	3.87	1.14	0.98	1.06	-0.04	-1.24	-1.06	-3.04	1.65	-1.11
**DNA_rich**	-0.09	0.96	-0.55	-1.03	-2.40	-0.44	-0.73	-1.15	0.51	-0.93
**SINE_ intermediate**	-0.05	-1.21	-0.32	-0.44	-4.14	-0.50	-0.74	0.96	-0.16	-1.13
**LINE_ intermediate**	0.31	3.69	0.81	0.39	-2.91	-2.58	-1.32	-2.55	0.44	0.62
**LTR_ intermediate**	7.73	7.76	0.25	-0.75	0.95	1.93	0.49	-0.45	-0.56	-0.04
**DNA_ intermediate**	3.98	-0.41	-0.45	1.61	-2.63	-0.14	-0.43	2.72	-0.17	-0.79
**all_TE_rich**	-2.75	1.08	-2.72	0.66	-3.35	-8.08	-0.66	-7.59	1.81	-1.10
**all_TE_intermedia te**	-0.15	0.52	-0.87	-1.09	-0.57	-0.75	0.20	-2.98	0.81	-0.75
**TE_free**	7.48	2.44	0.48	-2.46	0.06	7.36	0.84	-2.98	1.25	-1.32
**mix**	0.99	2.11	-0.92	-0.15	-1.49	-0.31	0.76	-2.12	0.51	-0.96
**P-values Kruskal Wallis tests**	4.60e-007	< 2.2e-16	2.15e-004	< 2.2e-16	< 2.2e-16	< 2.2e-16	5.53e-009	< 2.2e-16	< 2.2e-16	< 2.2e-16

Negative values indicate more enrichment in cancer condition
