# Supplementary material for: The transposable element environment of human genes is associated with histone and expression changes in cancer
Source: BMC Genomics. 2016 Aug 9;17:588. doi: 10.1186/s12864-016-2970-1 (PMC4979156; doi:10.1186/s12864-016-2970-1)
Supplement: Additional file 8: Table S6. — Most highly down-regulated TE-rich genes that are enriched for H3K27me3 in cancer condition (PDF 206 kb) [file 12864_2016_2970_MOESM8_ESM.pdf]

Table S6: most highly down-regulated TE-rich genes that are enriched for H3K27me3 in cancer condition

GO-Slim Biological process	Gene function	Gene name	Ensembl ID	Log2 Fold Change	H3K27me3 enrichment	
					Normal	Cancer
immune system process	Fc fragment of IgE, low affinity II, receptor for (CD23)	FCER2	ENSG00000104921	-11.60	1.28	6.29
response to interferon-gamma	2'-5'-oligoadenylate synthetase 2	OAS2	ENSG00000111335	-10.50	0	0.67
cellular process	chemokine (C-C motif) ligand 22	CCL22	ENSG00000102962	-10.01	0	4.30
immune system process	B-lymphocyte activation marker (BLAST-1) or signaling lymphocytic activation molecule 2 (SLAMF2) (IgSF superfamily) CD48 molecule	CD48	ENSG00000117091	-9.67	0	12.29
metabolic process	MX dynamin-like GTPase 1	MIX1	ENSG00000157601	-9.64	0.11	0.37
cellular process (blood coagulation)	tetraspanin 33	TSPAN33	ENSG00000158457	-9.48	0	5.82
B-cell mediated immunity	Epstein-Barr virus induced 3/interleukin-27 subunit beta	EBI3	ENSG00000105246	-9.10	0	2.57
cellular process	prepronociceptin	PNOC	ENSG00000168081	-8.97	0.73	6.26
response to interferon-gamma	2'-5'-oligoadenylate synthetase 1, 40/46kDa	OAS1	ENSG00000089127	-8.88	0	0.08
cation transport	mucolipin 2	MCOLN2	ENSG00000153898	-8.71	0	0.90
No GO annotation	amyloid beta (A4) precursor protein-binding, family B, member 1 interacting protein	APBB1IP	ENSG00000077420	-8.67	0	0.18
cellular process	toll-like receptor 10	TLR10	ENSG00000174123	-8.52	0	0.61
response to stress	Ras-related GTP binding D	RRAGD	ENSG00000025039	-8.40	0.57	6.38
**immune response**	**lymphocyte-specific protein tyrosine kinase**	***LCK**	**ENSG00000182866**	**-8.37**	**0**	**0.63**
cellular amino acid catabolic process	glycine dehydrogenase (decarboxylating)	GLDC	ENSG00000178445	-8.28	3.68	4.65

*gene identified as a cancer gene in the COSMIC database
