# Supplementary material for: The transposable element environment of human genes is associated with histone and expression changes in cancer
Source: BMC Genomics. 2016 Aug 9;17:588. doi: 10.1186/s12864-016-2970-1 (PMC4979156; doi:10.1186/s12864-016-2970-1)
Supplement: Additional file 9: Table S7. — Most up-regulated TE-rich and TE-intermediate genes that are enriched for H3K79me2 in cancer condition (PDF 213 kb) [file 12864_2016_2970_MOESM9_ESM.pdf]

Table S7: most up-regulated TE-rich and TE-intermediate genes that are enriched for H3K79me2 in cancer condition

GO-Slim Biological process	Gene function	Gene name	Ensembl ID	TE environment	Log2 Fold Change	H3K27me3 enrichment	
						Normal	Cancer
cation transport	Rh-associated glycoprotein	RHAG	ENSG00000112077	TE_intermediate	11.51	0	0.40
transcription from RNA polymerase II promoter	Rhox homeobox family, member 2	RHOXF2	ENSG00000131721		10.67	0	21.94
cellular process	Keratin 19	KRT19	ENSG00000171345		10.61	0	6.85
gamete generation, apoptotic process	Melanoma antigen family B, 2	MAGEB2	ENSG00000099399		10.56	0	8.78
No GO annotation	Serum deprivation response	SDPR	ENSG00000168497		10.30	0	3.26
**transcription from RNA polymerase II promoter**	**GATA binding protein 1 (globin transcription factor 1)**	**GATA1***	**ENSG00000102145**		**9.84**	**0**	**102.08**
metabolic process	DIRAS family, GTP-binding RAS-like 3	DIRAS3	ENSG00000162595		9.82	0	5.51
cellular process	Potassium voltage-gated channel, subfamily H (eag-related), member 2	KCNH2	ENSG00000055118		9.69	0	5.98
proteolysis	Cathepsin L	CTSL	ENSG00000135047		9.65	0	1.81
metabolic process	Aldehyde dehydrogenase 1 family, member A1	ALDH1A1	ENSG00000165092		9.60	0	3.01
system process transport	Hemoglobin, alpha 2	HBA2	ENSG00000188536		9.52	0	2.60
No GO annotation	Cadherin-like and PC-esterase domain containing 1	CPED1	ENSG00000106034		9.47	0	0.32
No GO annotation	Down syndrome critical region gene 8	DSCR8	ENSG00000198054		9.28	0	1.26
**transcription from RNA polymerase II promoter**	**GATA binding protein 2**	**GATA2***	**ENSG00000179348**		**9.04**	**0**	**2.98**
No GO annotation	Sclerostin domain containing 1	SOSTDC1	ENSG00000171243		8.83	0	0.80
polysaccharide metabolic process, protein glycosylation	UDP-N-acetyl-alpha-D-galactosamine:polypeptide N-acetylgalactosaminyltransferase 5 (GalNAc-T5)	GALNT5	ENSG00000136542		8.81	0	4.78
cellular protein modification process	Tumor suppressor candidate 3	TUSC3	ENSG00000104723		8.80	0	2.66
immune system process	Complement factor H	CFH	ENSG00000000971		8.78	0	1.36
cellular process	ADAM metallopeptidase with thrombospondin type 1 motif, 14	ADAMTS14	ENSG00000138316		8.58	0	0.49
No GO annotation	Glycophorin B (MNS blood group)	GYPB	ENSG00000250361		8.46	0	1.55
cellular process	N(alpha)-acetyltransferase 11, NatA catalytic subunit	NAA11	ENSG00000156269		8.29	0	7.42
cell cycle	Fibroblast growth factor 13	FGF13	ENSG00000129682		8.26	0	2.62
system process transport	Hemoglobin, alpha 1	HBA1	ENSG00000206172		8.24	0	42.80
No GO annotation	Selenium binding protein 1	SELENBP1	ENSG00000143416		8.21	0	3.06
fatty acid metabolic process	Annexin A1	ANXA1	ENSG00000135046		8.14	0	5.08
cell cycle	Peripheral myelin protein 22	PMP22	ENSG00000109099		8.13	0	0.71
cellular component movement	Filamin C, gamma	FLNC	ENSG00000128591		8.13	0	5.64

immune system process	Chemokine (C-X-C motif) ligand 3	CXCL3	ENSG00000163734		8.10	0	25.91
gamete generation, apoptotic process,	Melanoma antigen family B, 1	MAGEB1	ENSG00000214107		8.06	0	1.06
No GO annotation	Transmembrane protein 158	TMEM158	ENSG00000249992		8.06	0	4.45
transcription from RNA polymerase II promoter	Synovial sarcoma, X breakpoint 3	SSX3	ENSG00000165584	**TE_rich**	10.18	0	2.41
No GO annotation	Lin-28 homolog B (C. elegans)	LIN28B	ENSG00000187772		9.42	0	0.16
protein phosphorylation	Protein kinase, cAMP-dependent, regulatory, type II, beta	PRKAR2B	ENSG00000005249		9.42	0	7.11
immune system process	Hemoglobin, zeta	HBZ	ENSG00000130656		9.30	0	0.41
No GO annotation	Family with sequence similarity 178, member B	FAM178B	ENSG00000168754		9.19	0	1.76
response to stress	P antigen family, member 1 (prostate associated)	PAGE1	ENSG00000068985		9.19	0	16.44
immune system process	Protein S (alpha)	PROS1	ENSG00000184500		8.82	0	2.41
ion binding	Ret finger protein-like 4B	RFPL4B	ENSG00000251258		8.71	0	3.36
cellular protein modification process	ST6 (alpha-N-acetyl-neuraminyl-2,3-beta-galactosyl-1,3)-N-acetylgalactosaminide alpha-2,6-sialyltransferase 1	ST6GALNAC1	ENSG00000070526		8.50	0	0.67
response to stress	Meiosis specific with OB domains	MEIOB	ENSG00000162039		8.40	0	5.43
cellular protein modification process	Anti-Mullerian hormone receptor, type II	AMHR2	ENSG00000135409		8.35	0	1.75
anatomical structure formation involved in morphogenesis	Keratin 8	KRT8	ENSG00000170421		8.28	0	15.67
cytoskeletal protein binding	Myosin, light chain 4, alkali; atrial, embryonic	MYL4	ENSG00000198336		8.13	0	2.51

*genes identified as cancer genes in the COSMIC database
